# Tube Expansion Deformation Enables In Situ Synchrotron X-ray Scattering Measurements during Extensional Flow-Induced Crystallization of Poly l-Lactide Near the Glass Transition

**DOI:** 10.3390/polym10030288

**Published:** 2018-03-08

**Authors:** Karthik Ramachandran, Riccardo Miscioscia, Giovanni De Filippo, Giuseppe Pandolfi, Tiziana Di Luccio, Julia A. Kornfield

**Affiliations:** 1 Division of Chemistry and Chemical Engineering, California Institute of Technology, Pasadena, CA 91125, USA; kramacha@caltech.edu (K.R.); tidilu@caltech.edu (T.D.L.); 2 Division of Sustainable Materials, ENEA, Centro Ricerche Portici, 80055 Portici, Italy; riccardo.miscioscia@enea.it (R.M.); giuseppe.pandolfi@enea.it (G.P.); 3 Division of Photovoltaics and Smart Networks, Innovative Device Unit, Centro Ricerche Portici, 80055 Portici, Italy; giovanni.defilippo@enea.it

**Keywords:** PLLA, bioresorbable vascular scaffolds, stretch blow molding, biaxial elongation, SAXS, WAXS

## Abstract

Coronary Heart Disease (CHD) is one of the leading causes of death worldwide, claiming over seven million lives each year. Permanent metal stents, the current standard of care for CHD, inhibit arterial vasomotion and induce serious complications such as late stent thrombosis. Bioresorbable vascular scaffolds (BVSs) made from poly l-lactide (PLLA) overcome these complications by supporting the occluded artery for 3–6 months and then being completely resorbed in 2–3 years, leaving behind a healthy artery. The BVS that recently received clinical approval is, however, relatively thick (~150 µm, approximately twice as thick as metal stents ~80 µm). Thinner scaffolds would facilitate implantation and enable treatment of smaller arteries. The key to a thinner scaffold is careful control of the PLLA microstructure during processing to confer greater strength in a thinner profile. However, the rapid time scales of processing (~1 s) defy prediction due to a lack of structural information. Here, we present a custom-designed instrument that connects the strain-field imposed on PLLA during processing to in situ development of microstructure observed using synchrotron X-ray scattering. The connection between deformation, structure and strength enables processing–structure–property relationships to guide the design of thinner yet stronger BVSs.

## 1. Introduction

Coronary heart disease (CHD) results in obstructed blood flow to the heart due to the deposition of plaque on arterial walls. It is one of the leading causes of death in the world and claims over seven million lives each year [1,2,3]. Bioresorbable vascular scaffolds (BVSs) are emerging as a promising alternative to permanent metal stents for the treatment of CHD. These devices are referred to as “scaffolds” as opposed to “stents” owing to their transient nature in the implanted artery. In contrast to permanent metal stents, BVSs support the occluded artery for 3–6 months but are completely resorbed in 2–3 years [4], restoring vasomotion in the artery and minimizing the risk of fatal complications such as late stent thrombosis [5,6,7]. The first FDA-approved BVS [8] is made from the semicrystalline and biocompatible polymer poly l-lactide (PLLA), which hydrolyzes to form l-lactic acid, a metabolic product processed by the body [9,10,11]. However, PLLA is notorious for being a brittle polymer [12,13], making it a surprising choice for a device that must withstand crimping and deployment. PLLA blends [14,15] and copolymers [16,17] have superior ductility but these materials are not suitable for coronary implants as they prematurely lose strength due to rapid hydrolysis [18,19]. Therefore, processing conditions play a key role in overcoming PLLA’s inherent brittleness by providing the BVS with a microstructure that facilitates deployment and confers lasting radial strength. 

Bioresorbable vascular scaffolds are processed from extruded PLLA preforms in the following sequence: “tube expansion” biaxially stretches the initially amorphous preform and transforms it into a semicrystalline tube; “laser-cutting” converts the expanded tube into an as-cut scaffold that has a lattice network of struts; and “crimping” radially compresses the as-cut scaffold onto a balloon catheter for deployment in the artery [20]. The scaffold experiences severe local deformation during crimping (strain > 50%), yet shows no sign of failure upon deployment [20]. The strength of the scaffold in the deployed state has a counter-intuitive relationship with strength in the expanded tube. Tube expansion that is equibiaxial (200% strain in θ and 200% strain in *z*) creates a strong expanded tube; however, after laser-cutting and crimping, it performs poorly upon deployment (>40 cracks per scaffold when over-deployed) [20]. In contrast, tube expansion that is predominantly uniaxial (400% strain in θ and 20% strain in *z*) does not provide as strong an expanded tube, yet it produces scaffolds that perform well upon deployment (<10 cracks per scaffold when over-deployed) [20]. Microdiffraction results reconciled this disconnect in strength between the expanded tube and the deployed scaffold by elucidating the role of crimping; the predominantly uniaxial elongation process yields an expanded tube that develops a beneficial morphology in the crimped state that facilitates deployment without fracture [20]. The interplay of structural transformations that occur during tube expansion and crimping govern the strength of the deployed BVS, motivating investigation of structure development during the tube expansion process. 

The promising success of current 150 µm thick BVSs motivates the design of thinner scaffolds (~80 µm, similar to metal stents [21,22]) to extend the benefits of resorbable implants to a broader patient population. The key to a thinner BVS lies in careful control of its microstructure during processing so that an 80 µm BVS has strength comparable to the current 150 µm FDA-approved BVS. Tube expansion, the first step in the manufacture of a vascular scaffold, plays a central role in the design of thinner scaffolds. Similar to stretch blow molding, tube expansion subjects a PLLA preform to biaxial elongation near the glass transition inside an outer mold [23]. The rapid deformation induces oriented crystallization, which in turn influences the macroscopic strain and the wall thickness of the resulting BVS. The process of tube expansion has been applied with excellent reproducibility to manufacture scaffolds with uniform wall thickness that meet clinical standards. However, conventional methodology is unable to provide structural insight on tube expansion at time scales relevant to processing (>400% strain in a matter of seconds). As a result, tube expansion is largely based on trial-and-error; expanded tubes are subjected to mechanical characterization and process parameters are iteratively adjusted to optimize strength. 

Here, we discuss the implementation of a novel custom-built instrument that can subject polymeric preforms to tube expansion with acquisition of synchrotron X-ray scattering data at time scales relevant to processing (~1 s). To the best of our knowledge, the data acquired from this instrument are the first of their kind and relate deformation in the cylindrical geometry to the microstructure of the expanding preform. PLLA is subjected to tube expansion between 70 to 120 °C [24], relatively close to its glass transition (*T*g ~ 55 °C) and well below its melting temperature (*T*m ~ 170 °C). However, it is challenging to probe flow-induced crystallization in this temperature interval as PLLA is known to have poor thermal stability at elevated temperatures [25] and undergoes quiescent crystallization in the vicinity of 100 °C [26]. Therefore, a critical design requirement for our instrument is rapid heating—we are able to heat the preform above 100 °C (heating rate ~ 70 °C/min) and achieve inflation in less than 100 s; as a result, we avoid thermal degradation and isolate the impact of flow on the microstructure of the expanded tube. The primary emphasis of this article is on the morphology of PLLA, but the instrument and the insight gained from this approach can be broadly applied to study polymers (e.g., poly (ethylene terephthalate), PET) that are processed using stretch-blow molding. 

## 2. Materials and Methods

### 2.1. Design Considerations for the Tube Expansion Instrument

The design of our instrument builds upon the production protocol described in the patent literature for bioresorbable vascular scaffolds, specifically the tube expansion process described above [27]. Tube expansion uses compressed air to inflate a PLLA preform (inner diameter (ID): 0.64 mm, outer diameter (OD): 1.5 mm) inside a glass mold (ID: 3.9 mm, OD: 6.0 mm) at a desired temperature; the inflation transforms the initially amorphous preform into an oriented, semicrystalline tube. The mold dimensions are selected to limit the maximum expansion to an OD of 160% relative to the preform’s OD; in the present experiments, the expansion is self-arresting when the OD reaches approximately 150% of the preform’s OD. Infrared heating (IR) is used to provide a rise time of approximately 50 s from 35 to 100 °C, which is fast enough to avoid quiescent crystallization in the PLLA preform [26]. In contrast to hot air convection or restive heating, which both act on the mold, IR directly heats the preform. The choice of IR radiation motivates the use of borosilicate glass (Pyrex) for the mold as Pyrex is mostly transparent to IR wavelengths that are strongly absorbed by PLLA (see Supplementary Materials: Figure S1 defines the orientation of lamps relative to the mold and Figure S2 describes the energy absorbed by the preform relative to the mold). The instrument is designed to selectively vary processing parameters such as the expansion temperature (T^e^), the crystallization temperature (T^x^), the imposed pressure inside the tube during expansion (only one value is examined here, 6.8 bar) and the annealing conditions to isolate their impact on the resulting PLLA microstructure. 

### 2.2. Temperature Control

**Figure 1:**
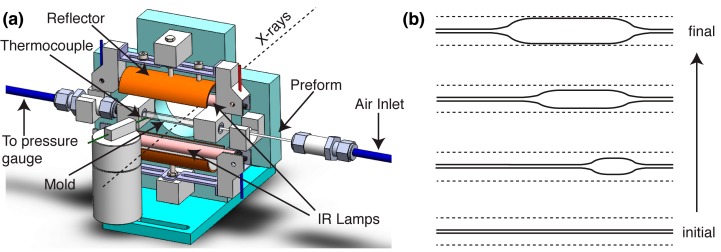
(**a**) A 3D diagram of the tube expansion instrument defines the positions of the IR lamps and reflectors above and below the mold, the thermocouple on the mold surface, the connection to the pressure transducer and the air inlet, and illustrates how the preform is inserted into the mold and oriented relative to the path of the X-rays. (**b**) Schematic diagram of the inflation process, which progresses from the side of tube attached to the air inlet to the opposite end.

The PLLA preform is heated above its glass transition temperature (Tg ~ 55 °C) using two 500 W Philips T3 halogen lamps (OD: 10 mm, length: 118 mm, Figure 1a) that are placed 25 mm on either side of the preform (Figure 1a). The lamps are operated using a 48 V power supply for user safety, which results in an effective power of ~100 W per lamp. The lamps are oriented parallel to the preform as this geometry minimizes gradients in temperature along the axial direction (~10° over 30 mm, Figure S1a). The lamps are surrounded by curved reflectors that improve the uniformity of heating and direct stray radiation towards the preform. Numerical simulations performed in Zemax indicate that the parallel orientation of lamps minimizes axial gradients in the absorbed energy (heat flux varies by ~10% along the 60 mm length of the preform, Figure S2) and that the reflectors enhance the rate of heating (the preform absorbs >200% more energy with the curved reflectors, Figure S2). The control box (described below) continuously records the temperature of the mold using a K-type thermocouple connected to a Maxim MAX6675 cold-junction compensated controller, which adjusts the power of the lamps to match the set heating rate and temperature. 

As the preform is expanded inside the Pyrex mold, it is challenging to experimentally probe the temperature of the preform. Therefore, we turn to numerical simulations that capture the geometry of the stretching apparatus to estimate the temperature of the PLLA preform. The volumetric heat flux provided by Zemax (~2.3 mW/mm^3^ for the mold and ~4.5 mW/mm^3^ for the preform, Figure S2) is incorporated in the finite element software Abaqus to predict spatial gradients in temperature for the mold and the preform as a function of time. The simulations predict a heating rate of ~1.1 °C/s for the mold and ~1.75 °C/s for the preform (Figure S3); the simulated heating rate of the mold is similar to experimental thermocouple data acquired on the mold (~1.2 °C/s, see Figure S3b, left for comparison). Furthermore, the simulations show that spatial gradients in temperature in the preform are ~1 °C after 50 s of heating (Figure S3a, right); a uniform temperature profile of the preform is consistent with the uniform thickness of the expanded tube (~140 µm). The higher temperature of the preform relative to the mold is corroborated by supporting experimental data, which also reveal that the preform temperature does not increase during the annealing step (Figure S4). 

### 2.3. Mechanical Assembly

The instrument core is comprised of a metal bracket that holds the IR lamps, the mold and the preform in position using Teflon supports (Figure 1a). The holders for the IR lamps are equipped with reflectors to direct stray radiation towards the mold-preform assembly (Figure 1a). A circular cutout in the metal bracket permits a path for X-rays to interact with the expanding preform (Figure 1a). Swagelok connectors secure one end of the preform to a source of compressed air (6.8 bar) and the opposite end to a Honeywell HSCDANN010BGAA5 pressure sensor (range 0–10 bar). The control box operates a pressure valve to trigger tube expansion, which propagates from the side of the tube attached to the air inlet towards the side connected to the pressure transducer (Figure 1b). 

### 2.4. Control Box

**Figure 2:**
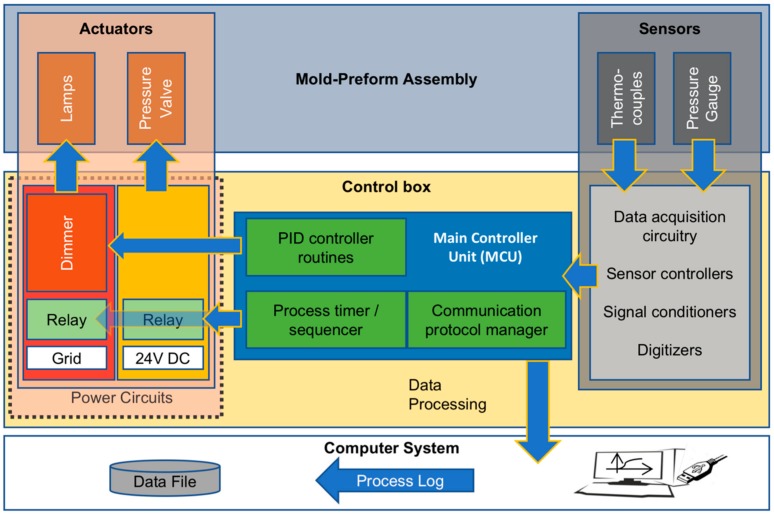
Block diagram describing the interface between the sensors, actuators, control unit and the data acquisition systems.

The control box is comprised of an Arduino Mega 2560 board (microcontroller unit, MCU) manufactured by Arduino, Italy that operates at a 16 MHz clock speed to acquire data from the sensors and to drive the actuators (Figure 2). The MCU uses a 5 V relay to operate the IR lamps and a numerically controlled dimmer to apply proportional-integral-derivative (PID) control to regulate the power of the lamps in accord with the set heating rate and temperature (Figure 2). A Parker Lucifer 341 L91 07 coil pressure valve is operated using a 24 V DC power supply and is controlled by the MCU via a 5 V relay to trigger expansion at user-defined setpoints (Figure 2). A Java-based desktop application issues commands to the control box and is responsible for plotting and logging data. 

### 2.5. Materials 

Clinical grade poly l-lactide (PLLA) preforms (ID: 0.64 mm, OD: 1.5 mm) were provided by Abbott Vascular, Santa Clara, CA, USA. 

### 2.6. In Situ Structural Characterization

The instrument was implemented at APS beamline 5-ID-D at the Argonne National Labs for the acquisition of simultaneous wide (WAXS) and small-angle X-ray scattering (SAXS) data. The incident X-ray beam is aligned parallel to the *r*-direction of the mold-preform assembly (Figure S5); diffraction patterns are acquired every 1 s at an exposure of 0.5 s using X-rays of wavelength = 0.7293 Å. WAXS and SAXS images were acquired on Rayonix CCD detectors with a sample to detector distance of 200.83 mm and 8.502 m, respectively. The wavevector *q* is calibrated using a spinning silicon diffraction grid. A subtraction method is implemented to isolate the scattering of PLLA (thickness of expanded tube ~ 140 µm) from that of the Pyrex mold (~1 mm thick) in the WAXS patterns (described in detail in the Supplementary Materials, Figures S6–S10). At small angles, there is little to no scattering from the Pyrex mold and the in situ SAXS patterns are presented as is (no subtraction). Video recordings from the beamline camera (Figure S5) permit estimation of the strain and strain-rate experienced by the PLLA preform during expansion.

## 3. Results

**Figure 3:**
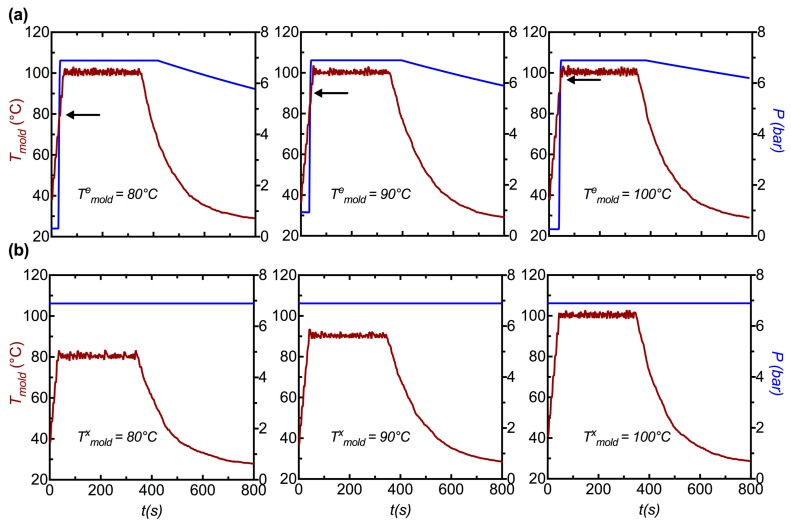
The transient temperature (red traces) and pressure (blue traces) profiles are presented in (**a**) for the first mode of operation and in (**b**) for the second mode of operation. In (**a**), inflation is triggered at a set mold temperature (*T^e^_mold_* = 80, 90 and 100 °C) and the tube is subsequently annealed at a mold temperature of 100 °C for 5 mins prior to cooling. In (**b**), the tubes are pressurized prior to heating (note the constant pressure trace) and instead the tubes are annealed at three different mold temperatures (*T^x^_mold_* = 80, 90 and 100 °C) for 5 mins prior to cooling. Numerical simulations described in the SI (Figures S2 and S3) predict that mold temperatures of 80, 90 and 100 °C translate to preform temperatures of ~ 105, 115 and 125 °C respectively.

Poly l-lactide (PLLA) cylindrical preforms are subjected to expansion using two modes of operation of the instrument. In the first mode, the pressure in the preform is not increased until a temperature of interest is reached during heating to a selected crystallization temperature: here, we use the temperature of the mold (*T*^e^_mold_ of 80, 90 and 100 °C, where superscript “e” denotes “expansion”) to trigger the elongation by opening a valve to 6.8 bar air, and use the mold temperature for feedback control during the isothermal crystallization process (*T*^a^_mold_ = 100 °C, where superscript “a” denotes “annealing”) for 5 min, followed by cooling under ambient conditions (Figure 3a). In the second mode of operation, the pressure in the preform is imposed prior to heating and is maintained throughout the process: here, we examine the effect of temperature during isothermal crystallization (*T*^x^_mold_ of 80, 90 and 100 °C, where superscript “x” denotes “crystallization”) for 5 min, followed by cooling under ambient conditions (Figure 3b). Numerical simulations predict that mold temperatures of 80, 90 and 100 °C correspond to PLLA preform temperatures of ~105, 115 and 125 °C, respectively (Figures S2 and S3). These two modes of operation were selected to isolate the impact of the expansion temperature (*T*^e^) from that of the crystallization temperature (*T*^x^) on the morphology of the expanded tube; expansion in the first mode is triggered at a set mold temperature while expansion in the second mode is reliant on the material properties of the preform.

**Figure 4:**
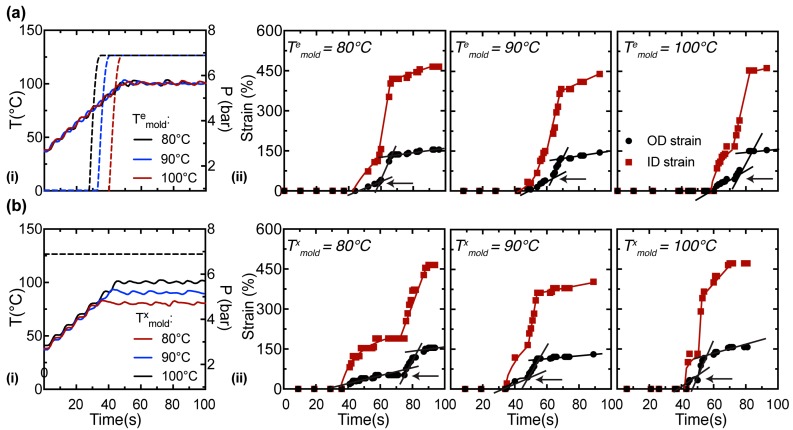
Plot of (**a**, **i**) temperature (solid lines) and pressure traces (dashed lines) for the first mode of operation; the pressure valve is triggered in ~5 s intervals to achieve expansion at *T^e^_mold_* = 80, 90 and 100 °C. The corresponding strain fields are presented in (**a**, **ii**). Plot of (**b**, **i**) temperature (solid lines) and pressure traces (dashed lines) for the second mode of operation; tubes expanded at *T^x^_mold_* = 80, 90 and 100 °C are pressurized to 6.87 bar prior to heating. The corresponding strain fields are presented in (**b**, **ii**). The strain at the outer diameter (OD, black circles in **a**,**b**, **ii**) of the preform is determined from video recordings (error in strain ~ 5%) and the strain at the inner diameter (ID, red squares in **a**,**b**, **ii**) is calculated from the OD strain assuming incompressibility (the difference in density between amorphous and crystalline PLLA is ~3% [28]). The slope of the straight black lines provides an estimate of the strain rate at the OD surface; the black arrows mark the inflection point when the strain rate abruptly increases. The error in synchronization between the video recordings and the synchrotron data (temperature, pressure and X-ray patterns) is ~5 s.

Video recordings are used to estimate the transient outer diameter (OD) of the preform, which changes in a complex manner with time (Figure 4). While some elongation may occur along the axial direction, it is much less than the hoop elongation. Here, we make the approximation of neglecting the axial elongation and analyze the outer diameter to estimate the strain at the outer surface. When the internal pressure is abruptly imposed at a prescribed temperature (Figure 4a, i), the initial deformation progresses at a similar rate (~0.025 s^−1^ at the OD, Figure 4a, ii) till an inflection point at an OD strain of 40–50% (marked by black arrows in Figure 4a, ii). After the inflection, the strain rate increases dramatically, to approximately 0.07 s^−1^ (Figure 4a, ii), until the deformation rate slows down when the OD strain approaches ~150%. The rates of deformation are similar in the first mode of operation (*T*^e^_mold_ = 80, 90 and 100 °C) as the mold temperature has reached a steady state (100 °C) during inflation. 

When the internal pressure is imposed before heating and maintained during heating (Figure 4b, i), the onset of deformation occurs when the PLLA preform reaches a temperature at which it can no longer withstand the pressure (Figure 4b, ii). This occurs approximately when the temperature of the mold reaches 80 °C. In the case of *T*^x^_mold_ = 80 °C, the mold has already reached a steady temperature when the PLLA begins to deform. On the other hand, for *T*^x^_mold_ = 90 and 100 °C, the temperature rises rapidly during the first several seconds of deformation, giving rise to progressively faster initial strain rates (the strain rate increases from 0.015 s^−1^ at *T*^x^_mold_ = 80 °C to 0.07 s^−1^ at *T*^x^_mold_ = 100 °C, t < 50 s in Figure 4b, ii). The initial deformation slows, giving a plateau prior to a steep increase in strain rate; the plateau OD strain is approximately 30–40% for all three values of *T*^x^_mold_, while the duration of the plateau decreases strongly with an increase in *T*^x^_mold_ (compare strain profiles in Figure 4b, ii). The rapid deformation at the end of the plateau rolls off and the final OD strain is approximately 150% for all three *T*^x^_mold_ cases. We now relate the macroscopic change in temperature and strain to the microstructure of the tube using in situ synchrotron X-ray scattering. 

**Figure 5:**
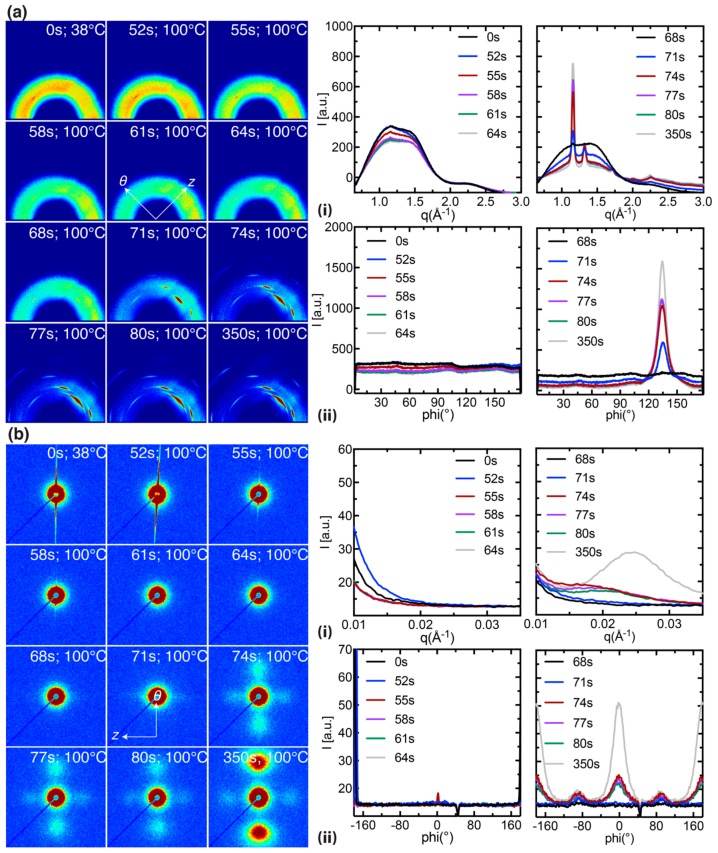
In situ (**a**) WAXS and (**b**) SAXS data acquired on a PLLA preform stretched at *T*^e^_mold_ = 100 °C. The diffraction patterns capture the transient heating and annealing steps (t < 350 s, *T*^a^_mold_ = 100 °C) and correspond to the temperature and strain profiles in Figure 3 and Figure 4a. Diffraction patterns acquired during the cooling step (t > 350 s) can be found in Figure S17. The WAXS and SAXS data are presented as: 2D patterns; (**a**,**b**, **i**) azimuthally averaged intensity I(q); and (**a**,**b**, **ii**) radially averaged intensity I(φ).

**Figure 6:**
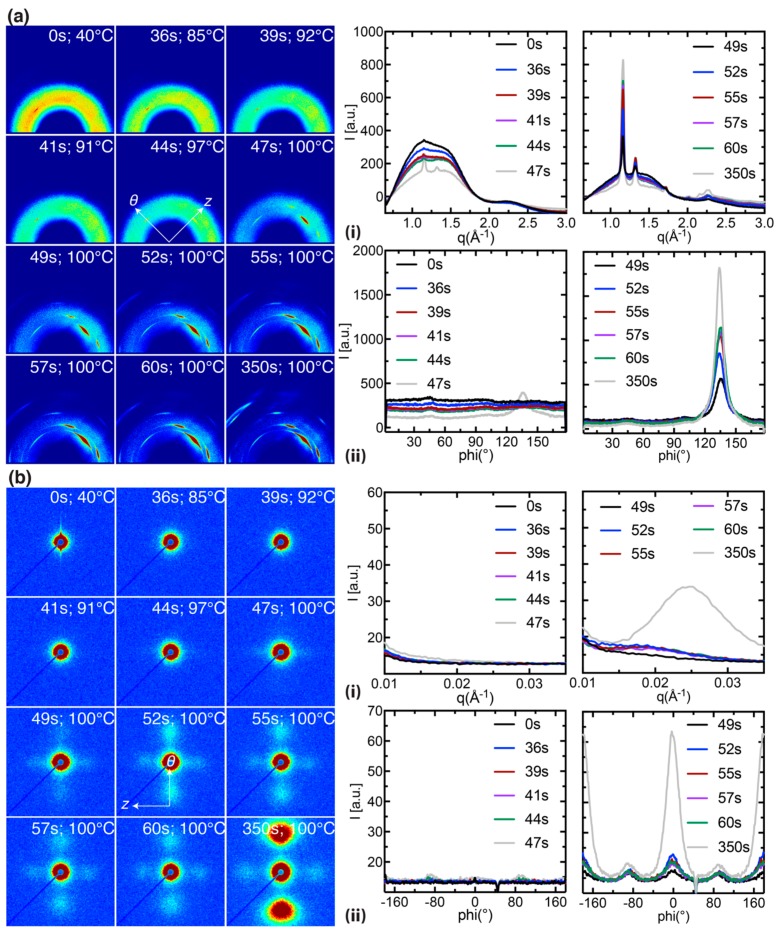
In situ (**a**) WAXS and (**b**) SAXS data acquired on a PLLA preform stretched at *T^x^_mold_* = 100 °C. The diffraction patterns capture the transient heating and annealing steps (t < 350 s, *T^a^_mold_* = 100 °C) and correspond to the temperature and strain profiles in Figure 3 and Figure 4b. Diffraction patterns acquired during the cooling step (t > 350 s) can be found in Figure S20. The WAXS and SAXS data are presented as: 2D patterns; (**a**,**b**, **i**) azimuthally averaged intensity I(*q*); and (**a**,**b**, **ii**) radially averaged intensity I(φ).

Here, we compare PLLA preforms stretched at *T*^e^_mold_ = 100 °C and *T*^x^_mold_ = 100 °C (in situ data for the other 4 experiments are presented in Figures S11–S14) to illustrate the impact of the onset of inflation (deformation occurs ~20 s later in *T*^e^_mold_ = 100 °C relative to *T*^x^_mold_ = 100 °C, Figure 4a,b, ii) on the morphology of the expanded tube. A selection of 12 diffraction patterns are presented for *T*^e^_mold_ = 100 °C and *T*^x^_mold_ = 100 °C that capture the transient expansion of the preform. Prior to expansion, we observe strong diffuse scattering from the amorphous phase and the presence of faint (110)/(200) peaks in the preform (compare Figure 5 and Figure 6a, 0 s); the azimuthal position of these peaks suggests that a small population of crystallites have their c-axis oriented parallel to the z-direction of the preform. It is likely that these z-oriented crystallites are created in the extrusion process used to prepare the preforms. The initial phase of inflation occurs at a relatively slow strain rate (~0.025 s^−1^ before the inflection point, Figure 4a, ii) and is characterized by a decrease in the amorphous intensity due to the thinning of preform; however, there is no indication of oriented crystallization in this interval (t < 70 s for *T*^e^_mold_ = 100 °C and t < 45 s for *T*^x^_mold_ = 100 °C, Figure 5 and Figure 6a). After the initial inflection in the strain field (black arrows in Figure 4a,b, ii), the strain rate suddenly increases to ~0.07 s^−1^ and is accompanied by distinct (110)/(200) peaks with their c-axis oriented parallel to the θ-direction of the preform (at ~68 s for *T*^e^_mold_ = 100 °C and ~44 s for *T*^x^_mold_ = 100 °C, Figure 5 and Figure 6a). The intensity of the newly formed θ-oriented (110)/(200) peaks increases rapidly over the next 10 seconds (68 to 77 s for *T*^e^_mold_ = 100 °C and 44 to 55 s for *T*^x^_mold_ = 100 °C; Figure 5 and Figure 6a), a time interval that corresponds to a > 50% increase in OD strain (compare the strain field in Figure 4a,b with WAXS data in Figure 5 and Figure 6a,b). Beyond 100 s, the expansion is nearly complete and amorphous material is steadily transformed into crystallites during annealing (Figure 5 and Figure 6a, 350 s). 

**Figure 7:**
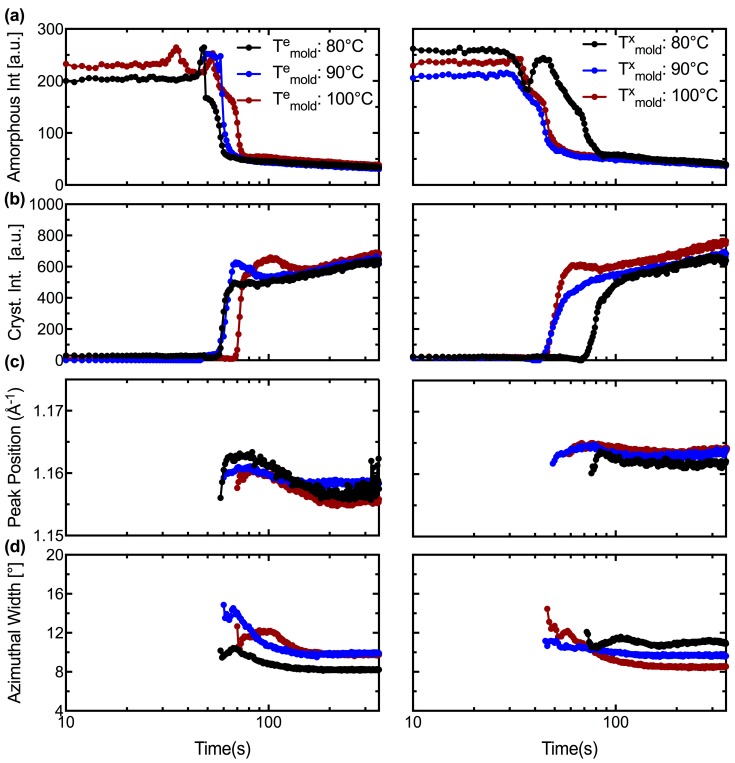
Quantitative characteristics of 1D WAXS profiles for expansion performed at: (**left**) *T^e^_mold_* = 80, 90 and 100 °C; and (**right**) *T*^x^_mold_ = 80, 90 and 100 °C. The variation in: (**a**) amorphous content; (**b**) crystallinity; (**c**) peak position of the (110)/(200) peaks; and (**d**) full width at half maximum of the (110)/(200) peaks is presented during the heating and the annealing steps (t ≤ 350 s); quantitative analysis for the cooling step is presented in the Supplementary Materials (Figure S21).

The azimuthal width of the (110)/(200) peaks (Figure 5 and Figure 6a, ii) provides a measure of crystallite orientation in a material. In the first mode of operation, expansion is triggered in ~5 s intervals at a mold temperature of 80, 90 and 100 °C (the pressure pulse requires ~10 s to reach 6.8 bar in the tube, see Figure 4a, i). However, the bulk of the expansion occurs when the mold temperature has reached 100 °C. Therefore, we observe similar rates of expansion and consequently, the azimuthal width of the oriented PLLA crystallites are similar (~8° for *T*^e^_mold_ = 80 °C and ~10° for both *T*^e^_mold_ = 90 and 100 °C, Figure 7d, left). In the second mode of operation, the tubes are pressurized prior to heating (Figure 4b, i) and it appears that the azimuthal width of the (110)/(200) peaks decreases monotonically as the crystallization temperature increases (~11° for *T*^x^_mold_ = 80 °C, ~9.5° for *T*^x^_mold_ = 90 °C and ~8.5° for *T*^x^_mold_ = 100 °C; Figure 7d, right). This seems counter-intuitive at first because the onset of inflation occurs at approximately the same time and temperature for all cases in the second mode of operation (Figures 3b and 4b). However, expansion progresses isothermally in the second mode of operation; therefore, the tube is expanding at a lower mold temperature in *T*^x^_mold_ = 80 °C relative to *T*^x^_mold_ = 100 °C and, consequently, the material deforms more rapidly at *T*^x^_mold_ = 100 °C (compare the strain rate for *T*^x^_mold_ = 80 °C vs. *T*^x^_mold_ = 100 °C for t < 60 s, Figure 4b, ii) and develops a microstructure with greater crystallite orientation. 

**Figure 8:**
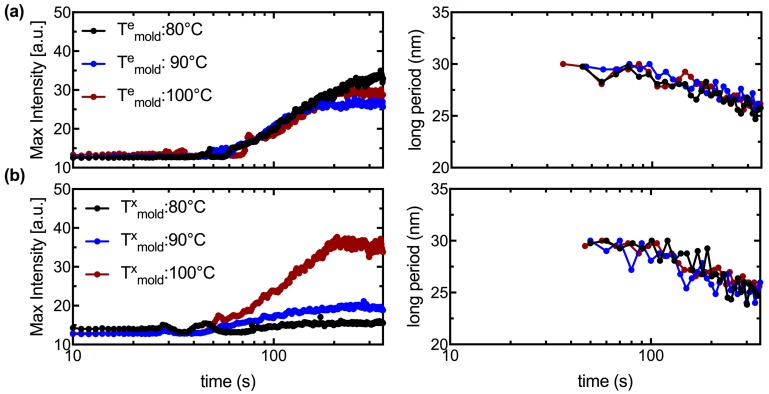
Quantitative characteristics of 1D SAXS profiles for expansion performed at: (**a**) *T^e^_mold_* = 80, 90 and 100 °C; and (**b**) *T^x^_mold_* = 80, 90 and 100 °C. The variation in: (**a**,**b**, **left**) the maximum intensity of the meridional SAXS peaks; and (**a**,**b**, **right**) the interlamellar spacing is presented during the heating and the annealing steps (t ≤ 350 s); quantitative analysis for the cooling step is presented in the Supplementary Materials (Figure S22).

Comparison between simultaneously acquired WAXS and SAXS patterns indicate that WAXS is more sensitive than SAXS to the onset of expansion (SAXS features appear a few seconds after the occurrence of (110)/(200) peaks in WAXS, compare Figure 5 and Figure 6a,b) [29]. The SAXS intensity continues to increase post expansion and adopts a bimodal distribution with peaks located along the meridian (parallel to the θ-direction) and the equator (parallel to the z-direction) of the patterns (see Figure 5 and Figure 6b, 350 s). The SAXS peaks along the meridian are more than twice as intense as the equatorial peaks, suggesting a dominant oriented microstructure along the θ-direction of the tube. The SAXS intensity decreases during the cooling step in accord with densification of the amorphous interlamellar space (t > 350 s, Figures S15–S20). Interestingly, both the equatorial and the meridional peaks shift towards higher-q during annealing, indicating a steady decrease in the long period (note the shift in peak intensity towards higher-q is more apparent in Figure 5 and Figure 6b, i; we return to this in the Discussion). The greater intensity of SAXS peaks in *T^x^_mold_* = 100 °C relative to *T^e^_mold_* = 100 °C (>15%, compare Figure 5 and Figure 6a,b, 350 s and Figure 8a,b, left) suggests that the former introduces a greater population of regular spaced lamellar stacks (>25 nm, Figure 8a,b, right) along the θ-direction of the expanded tube. 

**Figure 9:**
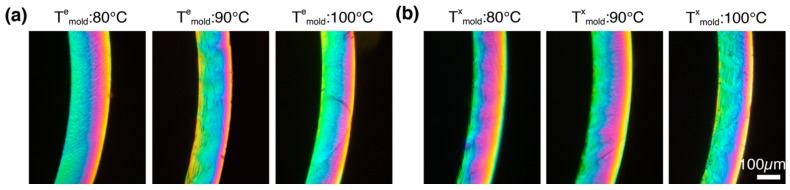
Expanded tubes stretched at: (**a**) *T*^e^_mold_ = 80, 90 and 100 °C (Figure 3 and Figure 4a); and (**b**) *T*^x^_mold_ = 80, 90 and 100 °C were extracted from the instrument and microtomed parallel to the (r,θ) plane to yield consecutive 50 µm thick circular cross sections. The 50 µm sections were sandwiched between glass slides and imaged through linear crossed polarizers. Note that the sequence of Michel Levy colors from blue green to purple to pink to orange to yellow unambiguously shows that the retardance decreases from ID to OD.

At the end of each experiment, the tubes are extracted from the instrument and are stored for ex situ studies. Polarized light micrographs of ~50 µm thick sections of the expanded tubes reveal a gradient in morphology from the inner (ID) to the outer diameter (OD): the retardance at the ID is ~1300 nm (third order bluish-green, Figure 9) and decreases steadily towards the OD (~800 nm, second order yellow, Figure 9). This gradient in retardance is not observed in the extruded tube and is a consequence of the tube expansion process, which imposes greater strains at the ID (>400%) relative to the OD (~150%). The greater orientation of PLLA chains at the ID relative to the OD manifests in the observed gradient in retardance.

## 4. Discussion

The tube expansion instrument provides structural information about PLLA during processing that is not captured by other techniques. The in situ WAXS patterns are very sensitive to the onset of inflation and probe changes in amorphous content, crystallinity, crystal morph, and crystallite orientation with a 1 s resolution. In all six experiments (Figure 3 and Figure 4), the deformation of the material occurs in two stages. During the first one, the material remains predominantly amorphous (Figure S11, 45–56 s; Figure S12, 49–58 s; Figure 5, 52–64 s; Figure S13, 45–65 s; Figure S14, 30–41 s; and Figure 6, 36–44 s). The second stage begins with an abrupt increase in crystallinity (Figure S11, 59 s; Figure S12, 62 s; Figure 5, 71 s; Figure S13, 75 s; Figure S14, 47 s; and Figure 6, 49 s), and a decrease in the amorphous halo (Figure 7a). This step increases in crystalline diffraction accounts for approximately half of the total increase in diffraction that occurs during deformation and subsequent annealing (Figure 7b). During the second process, strongly-oriented diffraction peaks grow with the chain axis along the θ-direction. These oriented WAXS and SAXS patterns suggest the presence of a “shish-kebab” morphology with shish along the θ-direction of the tube, as expected due to the large elongational strain (in excess of 400% at the inner diameter) imposed during tube expansion. This morphology is characterized by regularly spaced lamellar stacks called “kebabs” that decorate a central stem of oriented precursors called “shish” [30]. The sharp WAXS peaks are detectable earlier than the corresponding oriented SAXS peaks, with the delay being shorter when the sample is heated prior to imposing stress and with increasing temperature, becoming negligible for both *T*^e^_mold_ = 100 °C and *T*^x^_mold_ = 100 °C. Together, these features indicate that the initial process is dominated by glassy deformations and suggest that the transition to melt flow enables the combination of stretching, orientation and organization of the polymer chains that allows rapid growth of oriented crystals. This sequence accords with observations in the literature on poly (ethylene terephthalate) (PET) [29]. 

Specifically, our experiments are close enough to the glass transition that the initial deformation occurs at a strain rate that is faster than the rate of long range conformational rearrangement of the chains. In accord with Mahendrasingam [29], when only local segmental reorientation occurs, crystallization cannot. The second stage occurs on longer timescales, consistent with the expectation that relaxation of the chains allows orientation and stretch of submolecules that are long enough to participate in crystallization (dozens of consecutive units along the backbone). This corresponds to Mahendrasingam’s window of strain rate in which oriented crystals form during deformation, i.e., the strain rate is slow enough to permit chain retraction, but much faster than terminal relaxation by reptation. The transition between the two regimes correlates with the transition from glassy to rubbery mechanical properties.

Temperature affects the transition through its effect on the relaxation time, which is particularly strong for the glassy modes. The transition from glassy to rubbery relaxation offers a coherent explanation for the correlation between the abrupt increase in oriented crystallization (sharp WAXS peaks) and the abrupt increase in the rate of deformation (strain rates of ~0.07 s^−1^ or more after the inflection point, marked by black arrows in Figure 4a,b, ii). Experiments that use different crystallization temperatures suggest that the relaxation of the glassy modes requires tens of seconds at 80 °C, approximately 10–15 s at 90 °C and approximately 3–5 s at 100 °C (Figure 4b, ii). The rubbery relaxation is less sensitive to temperature, which explains the relatively similar rates of strain during the sudden tube expansion. It may also offer an explanation for the small effects of temperature on the final strain, manifested in the thickness of the expanded tube. There is precedent for flow-induced crystallization having such strong effects that the usual temperature dependence of crystallization is dramatically reduced [31]. Perhaps similar strain rates result in similar rates of oriented crystallization and, consequently, similar total strain when oriented crystallization brings deformation to a halt. 

An interesting and perhaps technologically important observation is the pronounced difference in the degree of orientation between PLLA expanded with the *T*^e^_mold_ = 80 °C and *T*^x^_mold_ = 80 °C protocols (cf. black symbols in Figure 7, left vs. right); the azimuthal width of the (110)/(200) peaks (Figure 5 and Figure 6a, ii) provides a measure of crystallite orientation. In the first mode of operation (Figure 7d, left), the preform is exposed to stress when the mold reaches *T*^e^_mold_ = 80 °C and heating continues until *T*^a^_mold_ = 100 °C. The glassy deformation stage is, consequently, brief and the rapid growth of oriented “kebabs” at 100 °C proceeds with strong correlation to the orientation of the “shish” created during elongation (see Figure S11 for X-ray data). In the second mode of operation (Figure 7d, right), the temperature at which expansion begins is dictated by the material properties as the stress is imposed on the tube throughout the heating process. In the *T*^x^_mold_ = 80 °C experiment (see Figure S13 for X-ray data), the sample begins to deform when the temperature of the mold reaches 80 °C and the deformation and crystallization processes take place isothermally at 80 °C. Due to the relatively low deformation temperature, there is a prolonged period of glassy deformation (approximately 20 s, Figure 4b,ii) when point-like precursors and threadlike precursors could form; and due to the relatively low crystallization temperature, the growth of crystallites from these precursors is slower and less correlated to the shish (Figure 7b, compare the slope of the main crystallization process for the black symbols). The dearth of oriented lamellar stacks for *T*^x^_mold_ = 80 °C is confirmed by SAXS results that show strong peaks indicative of shish-kebabs for *T*^e^_mold_ = 80 °C (black symbols in Figure 8a, left and Figure S11), but not for *T*^x^_mold_ = 80 °C (black symbols in Figure 8b, left and Figure S13). These results suggest that using a relatively low temperature during the tube expansion process could moderate the degree of anisotropy in the expanded tube, which is important for achieving balanced properties and ductile behavior in the BVS.

The intensity of the SAXS peaks increases during the first half of the annealing step (<200 s Figure 8a,b), then levels off. The increase in SAXS intensity occurs later and more gradually than the increase in WAXS (compare Figure 7b to Figure 8, left). This disconnect suggests that the increase in SAXS is due to a reorganization of previously formed crystals into increasingly coherent lamellae and, possibly, with a reduction of interlamellar density in favor of thicker crystallites (see Supplementary Materials for data on the cooling process, t > 350 s Figures S15–S20, where the SAXS intensity decreases). In conjunction with this reorganization, the interlamellar spacing monotonically decreases with time (Figure 8a,b, right). When this shift in long spacing occurs with an increase in SAXS intensity, it suggests that some interlamellar material is being pulled into the lamellae allowing the crystal to become wider. In some cases, the shift in long spacing occurs at a time when the SAXS intensity is decreasing, which suggests the growth of secondary lamellae in the space between previously formed lamellae. During the cooling step, the SAXS intensity of the present samples decreases due to the densification of amorphous interlamellar material and, possibly, growth of fringed micelles in the interlamellar space. By the time the sample temperature reaches ambient temperature, there is little to no evidence of lamellar superstructures (the SAXS pattern vanishes, see Supplementary Materials, t = 700 s in Figures S15–S20). Therefore, the transient structure reveals aspects of the present PLLA semicrystalline morphology that are concealed in the final state of the expanded tube. 

The tube expansion apparatus has several features that enabled these experiments. The device is small and relatively robust, making it well suited for transporting to a synchrotron. The device requires only a few connections, which enables rapid setup with minimal loss of beamtime. Infrared (IR) heating is rapid relative to sample ovens or heater blocks. Therefore, the samples reach the desired test temperature before deforming under their weight, enabling experiments relevant to stretch-blow molding. In the case of materials that undergo chemical reactions at elevated temperature, rapid heating can be used to minimize changes in the material prior to measurements.

The most significant area for improvement is in the synchronization of strain measurements with the in situ X-ray scattering data. Synchronization of video images could be accomplished by displaying a flash of light at selected time intervals. Strain measurements during intervals when the IR lamps are on could be accomplished by providing the camera with a high-pass optical filter (blocking red and infrared wavelengths). Improved quantification of the strain field could be accomplished by placing markings on the preform, provided the markings do not significantly absorb or scatter infrared light. Implementation of improved strain measurements would open the way to a whole space of biaxial elongations. Here, we were limited to constant width elongation, which can be evaluated from the transient radius of the tube. The apparatus already has provisions for superimposing axial elongation and azimuthal elongation. Faster strain rates can be examined if a faster camera is used. A broader range of stresses can be examined by varying the imposed pressure inside the preform or by simply modifying the dimensions of the preform. The instrument could be used with any of a number of in situ structural measurements (e.g., birefringence or light scattering) with appropriate modifications to offset lens effects of the curved interfaces of the mold and the samples. Therefore, the tube-expansion geometry may prove useful for the broad community of scientists who are investigating deformation induced structure in polymers and other soft matter.

## 5. Conclusions

Bioresorbable vascular scaffolds (BVSs) are manufactured from a nominally brittle polymer (PLLA) but gain strength and ductility through processing. Tube expansion plays a critical role in the manufacture of a BVS but defies prediction due to the rapid time scales of deformation. The selection of tube expansion conditions determines the final thickness of the scaffold and influences the response of PLLA during crimping and deployment. Therefore, a detailed understanding of the morphology created in the expanded tube holds the key to a thinner yet stronger scaffold. In this report, we discuss the fabrication of an instrument that can probe the structure of PLLA in real time at conditions relevant to the processing of vascular scaffolds. 

Video recordings and real time X-ray scattering data indicate that deformation in the PLLA preform progresses in two stages. During the first stage, PLLA exhibits glassy behavior and stretches at a relatively slow strain rate (0.03 s^−1^) until the strain at the outer diameter (OD) reaches an inflection at ~50%. In the second stage, PLLA exhibits rubbery behavior (OD strain > 50%) and the strain rate abruptly increases (0.07 s^−1^) till an OD strain of ~130%. The in situ WAXS data indicate that the material is predominantly amorphous during the first stage of deformation but develops oriented crystallites at the onset of the second stage of deformation. The corresponding SAXS data suggest that the rapid inflation induces growth of shish-kebabs along the θ-direction of the tube. By varying process parameters such as the expansion temperature, (*T*^e^_mold_), the crystallization temperature (*T*^x^_mold_), and the annealing conditions, we demonstrate that the microstructure of the expanded tube can be controlled to vary the crystallinity, crystallite orientation and the population of shish-kebabs to obtain desired mechanical properties. 

The data presented in this report highlight the ability of a tube expansion instrument to provide in situ X-ray scattering data during and after elongational deformation. Future studies will probe the impact of total strain by using molds of different dimensions and strain-rate by varying the inflation pressure. The combination of in situ structural data with the transient strain field may prove broadly useful for understanding processing–structure–property relationships in polymers, as illustrated by the example of PLLA relevant to bioresorbable vascular scaffolds that may improve treatment of cardiovascular disease. 

## Supplementary Materials

The following are available online at http://www.mdpi.com/2073-4360/10/3/288/s1, Figure S1: Selection of lamp orientation relative to the preform, Figures S2 and S3 & Table S1: Numerical simulations of the temperature profile of the preform, Figure S4: Experimental data used to validate the simulations, Figure S5: Implementation of the instrument at an X-ray beamline, Figures S6–S10: X-ray scattering analysis, Figures S11–S20: Summary of in situ WAXS and SAXS data, Figures S21 and S22: Quantitative analysis of in situ WAXS and SAXS data.
