# Supplementary material for: Tube Expansion Deformation Enables In Situ Synchrotron X-ray Scattering Measurements during Extensional Flow-Induced Crystallization of Poly l-Lactide Near the Glass Transition
Source: Polymers (Basel). 2018 Mar 8;10(3):288. doi: 10.3390/polym10030288 (PMC6415077; doi:10.3390/polym10030288)
Supplement: Supplementary file 1 [file polymers-10-00288-s001.pdf]

polymers

Article – Supporting Information

Tube expansion deformation enables *in situ* synchrotron x-ray scattering measurements during extensional flow-induced crystallization of poly L-lactide near the glass transition

Karthik Ramachandran [1], Riccardo Miscioscia [2], Giovanni De Filippo [3], Giuseppe Pandolfi [2], Tiziana Di Luccio [1,2] and Julia A. Kornfield [1]*

[1] Division of Chemistry and Chemical Engineering, California Institute of Technology, Pasadena, CA 91125, USA; kramacha@caltech.edu (K.R.); tidilu@caltech.edu (T.D.L.); jak@cheme.caltech.edu (J.A.K)
[2] Division of Sustainable Materials, ENEA, Centro Ricerche Portici, 80055 Portici, Italy; riccardo.miscioscia@enea.it (R.M.); giuseppe.pandolfi@enea.it (G.P.)
[3] Division of Photovoltaics and Smart Networks, Innovative Device Unit, Centro Ricerche Portici, 80055 Portici, Italy; giovanni.defilippo@enea.it
* Correspondence: jak@cheme.caltech.edu; Tel.: +1-626-395-4138

Received: 11 February 2018; Accepted: 6 March 2018; Published: date

Supporting Information

Selection of lamp orientation relative to the preform

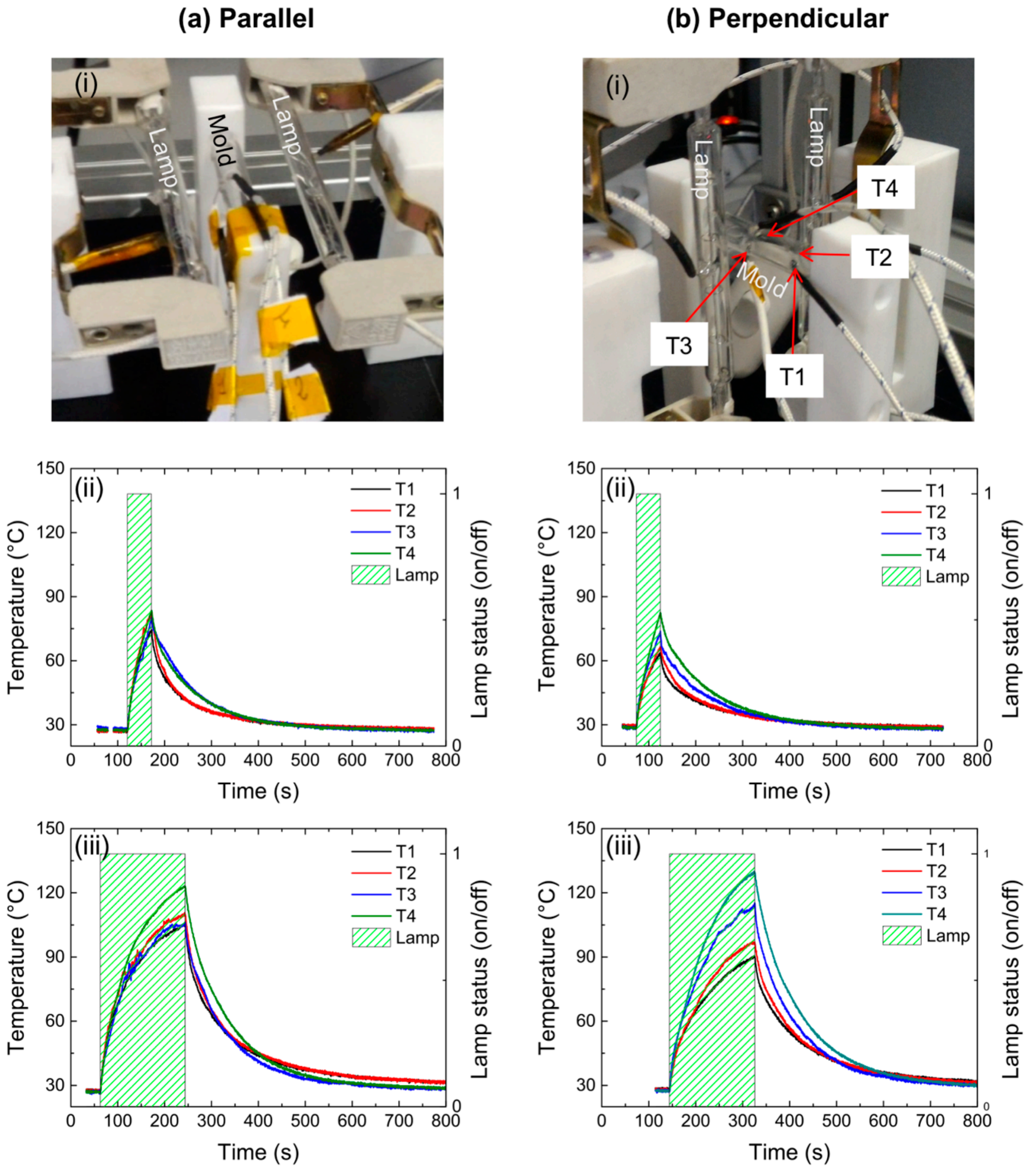

Figure S1: Four thermocouples (T1 to T4) are placed at different positions along a customized Pyrex mold to probe azimuthal and axial gradients in temperature induced by the IR lamps oriented **(a)** parallel to the preform and **(b)** perpendicular to the preform. T1 and T2 are located ~30mm from the center of the mold while T3 and T4 are at the center of the mold. T1 and T3 probe the temperature of the mold surface directly facing the lamps while T2 and T4 probe the temperature of the mold surface 90° from T1 and T3. Temperature traces are plotted for a lamp exposure time of (a-b, ii) 50s and (a-b, iii) 180s.

It is desirable to achieve uniform heating of the preform prior to expansion for homogeneous deformation and wall thickness. The IR lamps (OD:10mm and length:118mm) can be oriented either parallel or perpendicular to the preform. We tested both configurations (Fig. S1a-b,i) using a customized Pyrex mold (ID: 8mm, OD:10mm and length: 60mm) with incisions for thermocouples made at the center (one facing the lamps and the second 90° away) and 30mm from the center (one facing the lamps and the second 90° away). For an exposure time of 50s, the difference in temperature

across all four thermocouples is ~10°C for the parallel configuration but is ~20°C for the perpendicular configuration (compare Fig.S1a-b, ii); prolonged exposure (180s) increases these gradients to ~15°C for the parallel configuration and >30°C for the perpendicular configuration (compare Fig. S1a-b, iii). It is reasonable to expect that the parallel configuration minimizes axial gradients in temperature as the lamps illuminate a greater portion of the mold surface. Based on these data, we orient the lamps parallel to the mold for the experiments described in this report.

Numerical simulations of the energy absorbed by the mold and the preform

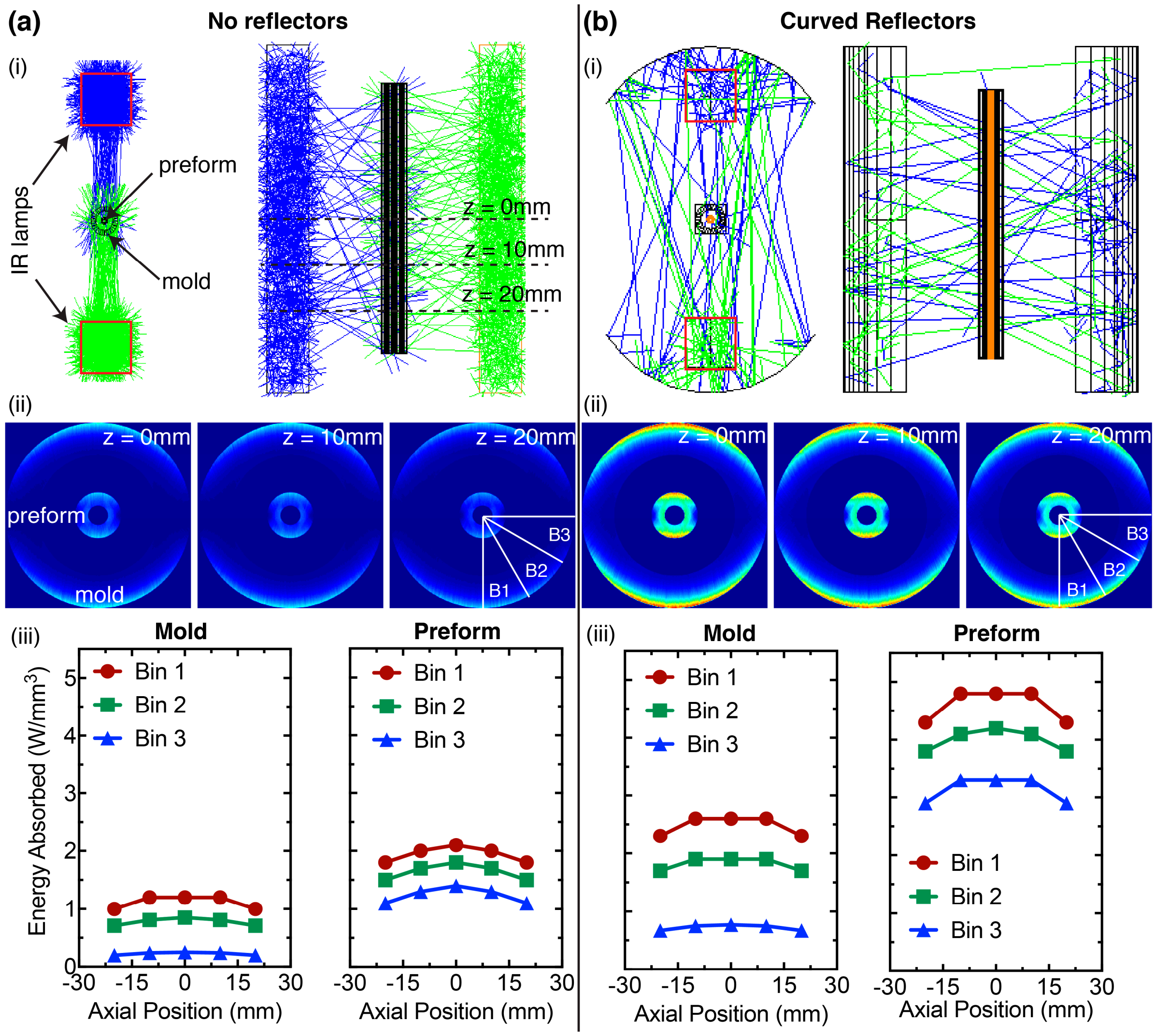

Figure S2: Ray tracing calculations performed in Zemax estimate the fraction of Infra-red (IR) light absorbed by the mold and the preform **(a)** without reflectors and **(b)** with curved reflectors. The fraction of energy absorbed is presented as **(a-b, ii)** 2D maps at different positions along the axis of the mold and the preform. The 2D data are averaged to calculate **(a-b, iii)** from the inner to the outer diameter to obtain the azimuthal distribution of absorbed energy (bin size is 30°) for the mold **(left, a-b, iii)** and the preform **(right, a-b, iii)**; Bin 1 is facing the lamps and Bin 3 is 90°C from the plane containing the lamps.

The two 500W IR lamps have an operating temperature of 2900K; Planck's law is used to estimate the fraction of light absorbed by Pyrex (the mold) and poly L-lactide (PLLA, the preform) in accord with their IR absorption spectra [1]. Pyrex is mostly transparent to IR radiation between 1 to 2.7μm (Table S1), a region where PLLA strongly absorbs IR radiation (particularly between 2.2 to 2.7μm, Table S1). Beyond 3μm, Pyrex absorbs most of the IR radiation and hardly any light passes through (Table S1). We estimate that for a 1mm thickness, the mold and the preform absorb ~15% and ~14% of incident IR radiation respectively (Table S1).

The fraction of energy absorbed by Pyrex and PLLA guide the setup of ray tracing simulations in Zemax to determine the distribution of energy in the perform and the mold (Fig. S2). The preform (ID: 0.64mm, OD: 1.52mmm, length: 60mm) is placed inside the mold (ID: 3.9mm, OD: 6.0mm, length: 60mm) and the two IR lamps (OD: 10mm, total length: 118mm, filament length: 82mm) are positioned 25mm on either side of the preform (Fig. S2, i) in the parallel configuration (described in Figure S1).

As the two 500W IR lamps are operated at 48V, they are assigned an effective power of ~100W in Zemax (of this, it is assumed that the mold can absorb a maximum of 15W and the preform can absorb a maximum of 14W). Ray tracing calculations are performed with and without curved reflectors (arc length: 42mm, axial length: 82mm, radius: 25mm), placed 10mm from the OD of the lamps, to note their impact on the absorbed radiation (Fig. S2, i).

Table S1: Fraction of incident IR radiation absorbed by the Pyrex mold and the PLLA preform

Wavelengths	Fraction of Total Energy	Fraction absorbed by Pyrex mold	Fraction absorbed by PLLA preform
0µm - 1.5µm	48%	<5%	<5%
1.5µm - 2.2µm	24%	<5%	20%
2.2µm - 2.7µm	10%	<5%	95%
2.7µm – 3µm	4%	50%	~0%
3µm – 3.5µm	3.5%	54%	~0%
3.5µm – 4µm	2.5%	75%	~0%
>4µm	10.5%	>90%	~0%

The simulations provide insight on the distribution of energy with and without reflectors along the axial and azimuthal directions of the mold/preform. The reflectors induce a 200% increase in the energy absorbed by the PLLA preform (compare a-b, Fig. S2).; the heat flux in Bin 1 (B1), a volume element directly facing lamps, increases from ~ 2.1 mW/mm^3 to ~4.8 mW/mm^3 (compare absorbed flux for preform in Fig. S2a-b, ii-iii). An increase in the absorbed IR radiation translates to a faster heating of the preform, which is desirable to minimize quiescent crystallization prior to expansion. The simulations indicate minimal gradients in the absorbed energy along the z-axis (~10% from the center to the edge, Fig. S2, ii-iii) but suggest substantial gradients along the azimuthal direction (>30%, for both the mold and the preform from Bin 1 to Bin 3, which is facing away from the lamps; Fig. S2, iii).

The heat flux data from Zemax are used in the finite-element software Abaqus to estimate the rate and distribution of temperature in the mold (ϱ = 2.2 g/cm^3, k = 1 W/m-K, Cp = 750 J/kg-°C [2]) and the preform (ϱ = 1.3 g/cm^3 [3], k = 0.13 W/m-K [4], Cp = 1800 J/kg-°C [5]). Abaqus predicts that the PLLA preform heats up faster than the mold: after 50s of heating, the mold reaches a temperature of ~90°C (rate: ~1.1°C/s, Fig. S3) while the preform reaches a temperature of ~125°C (~1.75°C/s, Fig. S3). Furthermore, the azimuthal gradients for both the mold and the preform are minimal (<3°C, Fig. S3), suggesting that there are no "hot spots" in the preform that can lead to non-uniform expansion. The faster heating rate of the preform is in agreement with experimental data (Fig. S4). The instrument was modified to probe the temperature of the mold, a PLLA preform inside the mold, and a PLLA preform outside the mold during the heating and annealing steps (Fig. S4a); for a set mold temperature of 85°C, the PLLA preform inside the mold is ~20°C warmer at the onset of annealing (Fig. S4b). Furthermore, the PLLA preform temperature does not increase during the annealing step (Fig. S4b), indicating that the rapid increase in strain beyond the inflection point (Fig. 4a,b,ii) is not influenced by an increase in temperature.

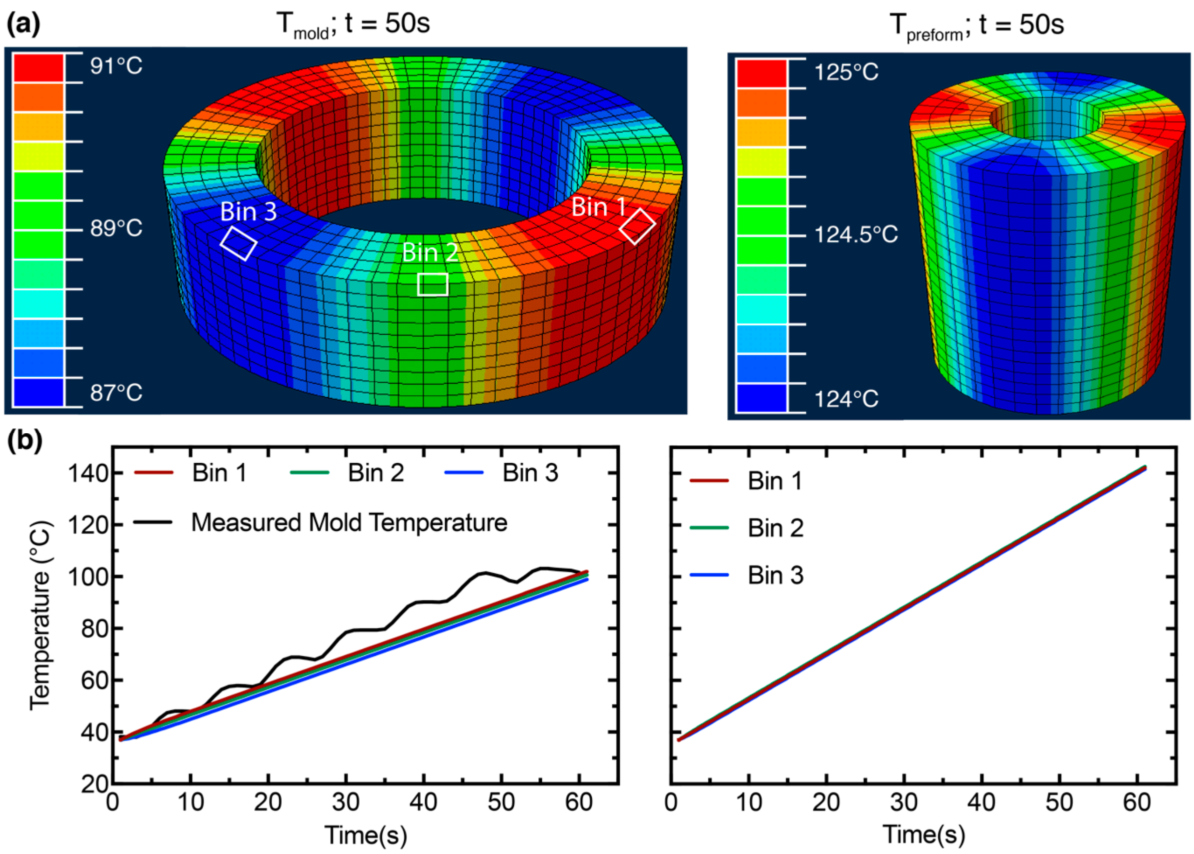

Figure S3: (a) 2D heat map generated by Abaqus illustrating the azimuthal gradients in temperature for the mold (left) and the preform (right) during heating for 50s. **(b)** 1D plots for bins located 0, 45 and 90° from the plane containing the lamps (location of bins defined in a,left) for the mold (left) and the preform (right). The predicted temperature of the mold (bins 1-3 in b, left) is compared with experimental data acquired on the surface of the mold (black line in b, left).

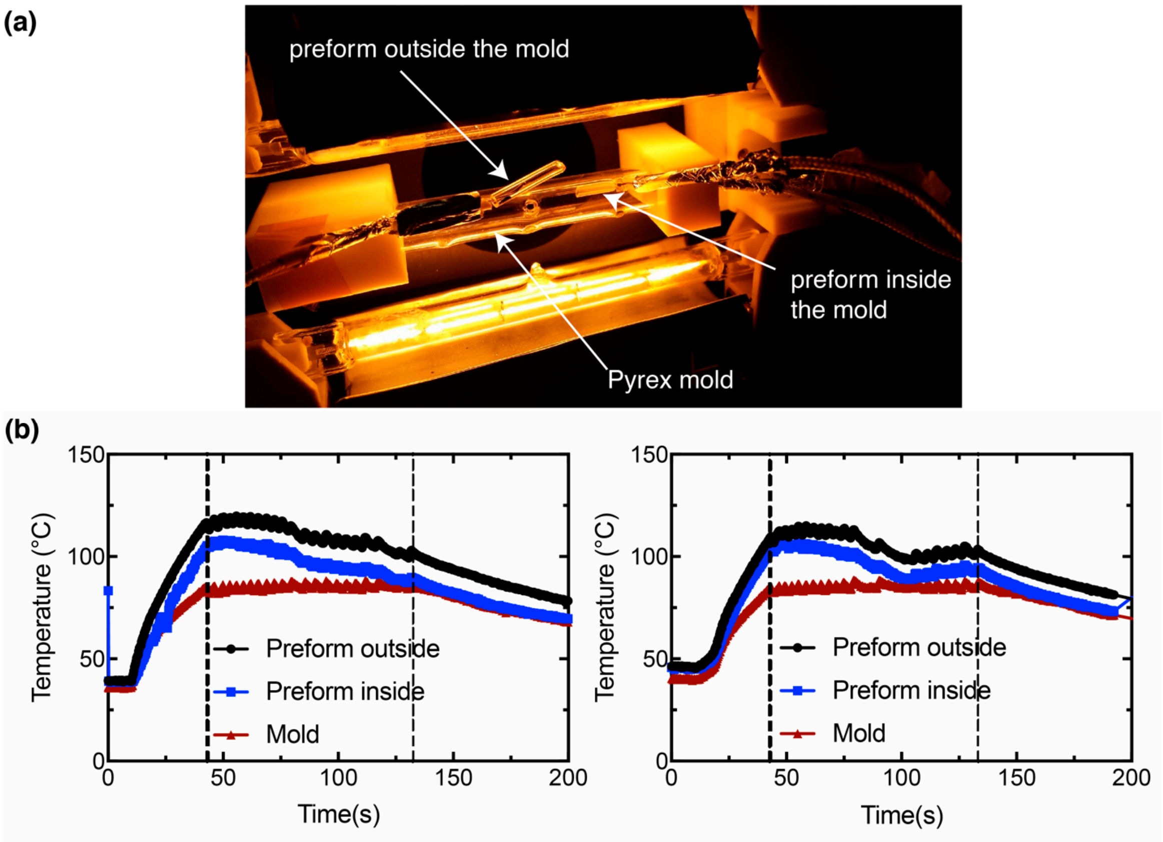

Figure S4: The instrument is modified to simultaneously probe the temperature of the mold in relation to a PLLA preform both inside and outside the mold. **(a)** image highlighting the position of the preforms with respect to the mold. **(b)** Temperature traces for the preforms and the mold are presented for two consecutive cycles of heating and cooling (b, left and right). The first dashed vertical line indicates the onset of annealing (at a set mold temperature of 85°C) and the second dashed vertical line indicates the end of annealing and the onset of cooling.

Implementation of the instrument at the X-ray beamline

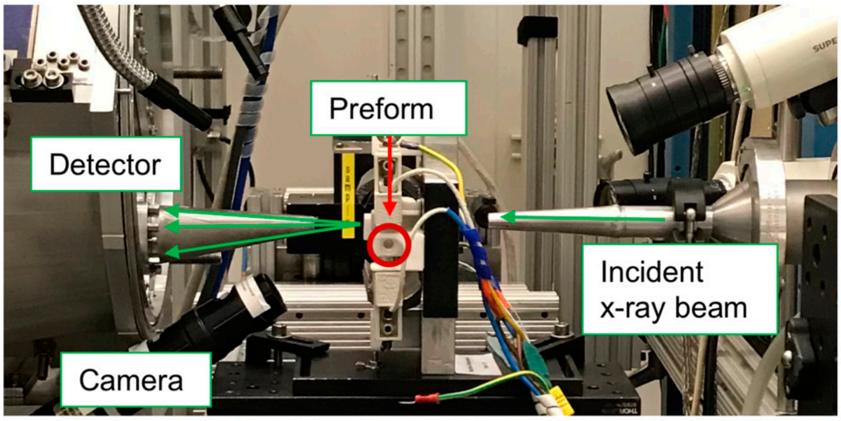

Figure S5: Position of the instrument with respect to the incident X-ray beam, the detectors (only WAXS detector is visible here) and the camera used to monitor inflation. The preform, molds and IR lamps are placed with their z-axis normal to the plane of the image.

The instrument is mounted on an optical bench at the beamline that can be translated in the horizontal direction (in and out of this plane) and the vertical direction (up and down in this plane) for sample alignment. The instrument is placed between two nose cones (identified by green arrows, Fig. S5) that are 70mm apart. Incident X-rays travel from the right nose cone and scattered X-rays are probed by detectors towards the left (Fig. S5). It is critical to avoid contact with the nose cones when the instrument is mounted/dismounted from the beamline. The instrument was designed with these spatial constraints in mind to have a minimal footprint at the synchrotron beamline.

X-ray scattering analysis

Poly L-lactide (PLLA) performs are expanded inside Pyrex molds with simultaneous acquisition of wide (WAXS) and small angle X-ray scattering (SAXS) data. The thickness of PLLA in the beam varies from ~400μm (preform) to ~140μm (expanded tube), which is less than half that of the Pyrex mold (~1mm). As a result, relatively strong scattering from the mold obscures WAXS features of the expanded tube, particularly in the vicinity of q ~1.5 Å^{-1} (Fig. S6). Diffraction patterns acquired on the mold alone indicate a ~15% variation in the scattered intensity (~300 counts, Fig. S7), which is ~50% of the scattering from PLLA alone (~700 counts, Figs. 5-6 and Figs. S11-S14). Therefore, direct subtraction of the Pyrex background from PLLA+Pyrex frames results in under or over-subtraction. The variation in scattered intensity of the mold can be attributed to a shift in the position of the mold with respect to the beam from one experiment to the other; this subtle variation in the thickness of the mold gives rise to discrepancies during subtraction. To overcome this complication, we take an average in 2D of 30 Pyrex patterns acquired immediately before and after tube expansion experiments over a period of 7 hours; this average background frame is then rescaled using the method described below and subsequently subtracted from PLLA+Pyrex frames to minimize subtraction errors.

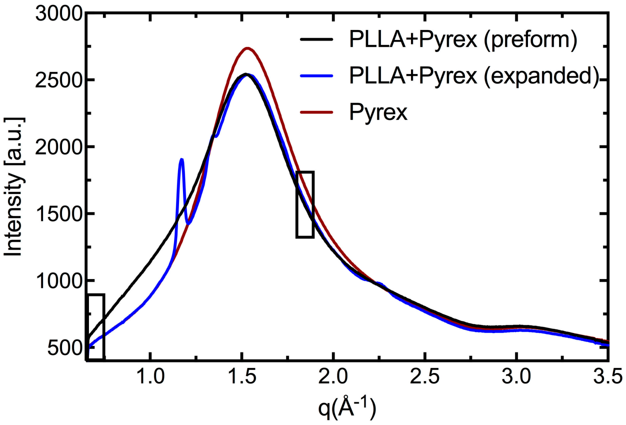

Figure S6: Comparison between 1D WAXS profiles for PLLA+Pyrex (before and after expansion) and the Pyrex mold.

We rescale the background by identifying q-intervals for the PLLA preform that are mostly unchanged before and after expansion. The prior literature informs us that scattering from PLLA is negligible at a low-q interval of 0.5-0.6 Å^{-1}. However, we do not have access to scattering below 0.68 Å^{-1} at this beamline due to the size of the beamstop. Therefore, the lowest possible q-interval available to us is 0.68 to 0.75 Å^{-1} (indicated by a black box in Fig. S6); the intensity in this q-interval changes during inflation (t < 100s, Fig. S8a) but hardly varies post expansion (t > 100s, Fig. S8a). At high-q, we use an interval spanning 1.80 to 1.90 Å^{-1} (indicated by a black box in Fig. S6) as the intensity in this region varies by ~ 3% before and after inflation (Fig. S8b). Therefore, we use the average intensity in these low-q (0.68 to 0.75 Å^{-1}) and high-q (1.80 to 1.90 Å^{-1}) intervals to two define two parameters (α and β) to rescale the average Pyrex background with respect each PLLA+Pyrex frame. The parameter α relates the difference in intensity (ΔI) between the low-q (I_L) and high-q (I_H) intervals for the PLLA+Pyrex frame to that of the average Pyrex frame. The parameter β applies an offset to the average Pyrex background to match the I_H of the PLLA+Pyrex frame. Using q for the radial wavevector and φ for the azimuthal angle, we arrive at the following equations to isolate the scattering of PLLA ($S_{subtracted}$) from PLLA+Pyrex (S) by subtracting the average Pyrex frame (B):

$$S(q,\varphi)_{subtracted} = S(q,\varphi) - \alpha . B(q,\varphi) - \beta \qquad [S1]$$

$$\alpha = \frac{I_L^S - I_H^S}{I_L^B - I_H^B} \qquad [S2]$$

$$\beta = I_H^S - \alpha\, I_H^B \qquad [S3]$$

The two-parameter subtraction method is applied in 2D to rescale the background, which is subsequently subtracted pixel by pixel from the frame of interest. To test our approach, the rescaled background is subtracted from each of the Pyrex frames in Figure S7 and the resulting residuals (Fig. S9) are analyzed in relation to the scattered intensity from PLLA alone. The impact of rescaling on the averaged background is illustrated in Figure S10; the shape of the rescaled background (dashed golden line, Fig. S10) mimics that of each Pyrex frame (black line, Fig. S10). As a result, the resulting error in subtraction (~20 counts, Fig. S9) is < 3% of the scattered intensity from expanded PLLA (~700 counts, Figs. 5-6 and Figs. S11-S14). We acknowledge that this method introduces errors in subtraction at low-q (0.68 to 0.75 Å^{-1}) for PLLA frames prior to expansion, but these errors do not affect our interpretation of the data.

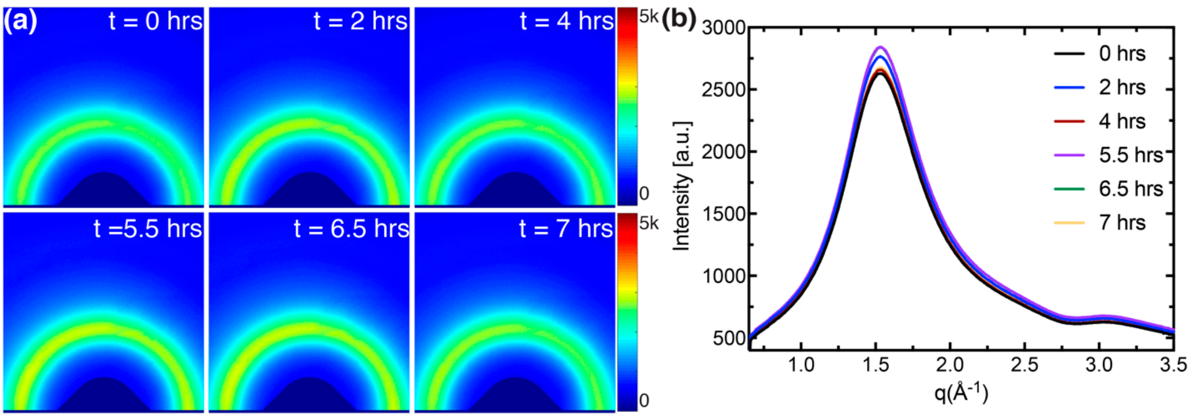

Figure S7: Variation in scattering from the Pyrex mold over a period of 7 hours during tube expansion experiments. The WAXS data are presented as **(a)** 2D patterns and **(b)** azimuthally averaged, I(q), 1D plots.

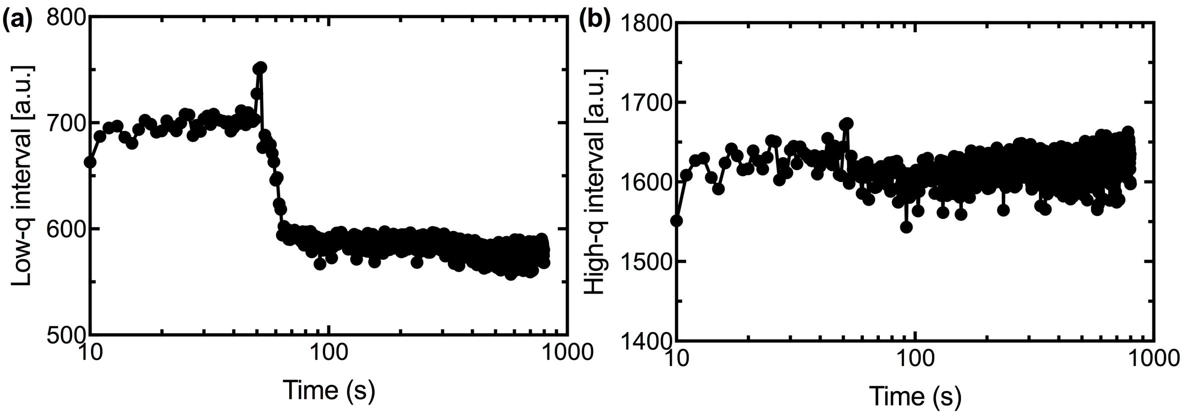

Figure S8: Drift in the average intensity at **(a)** low-q (0.68 to 0.75 Å^{-1}) and **(b)** high-q (1.80 to 1.90 Å^{-1}) for T^{e}_{mold} = 80°C (Fig. S10) during tube expansion. The sudden decrease in the low-q intensity at ~50s corresponds with inflation of the PLLA preform (the decrease in thickness of the tube and the transformation of amorphous content to crystallites is responsible for the drop in amorphous content at low-q).

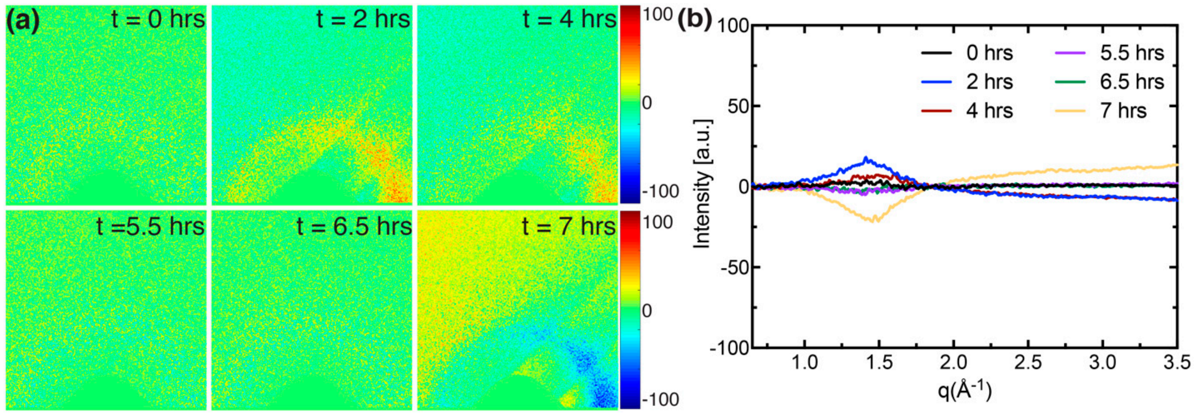

Figure S9: The error is subtraction is minimal when the average Pyrex background is rescaled using the two-parameter method and subtracted from each of the Pyrex frames (see Fig. S2). The residuals are presented as **(a)** 2D patterns and **(b)** azimuthally averaged, I(*q*), 1D plots.

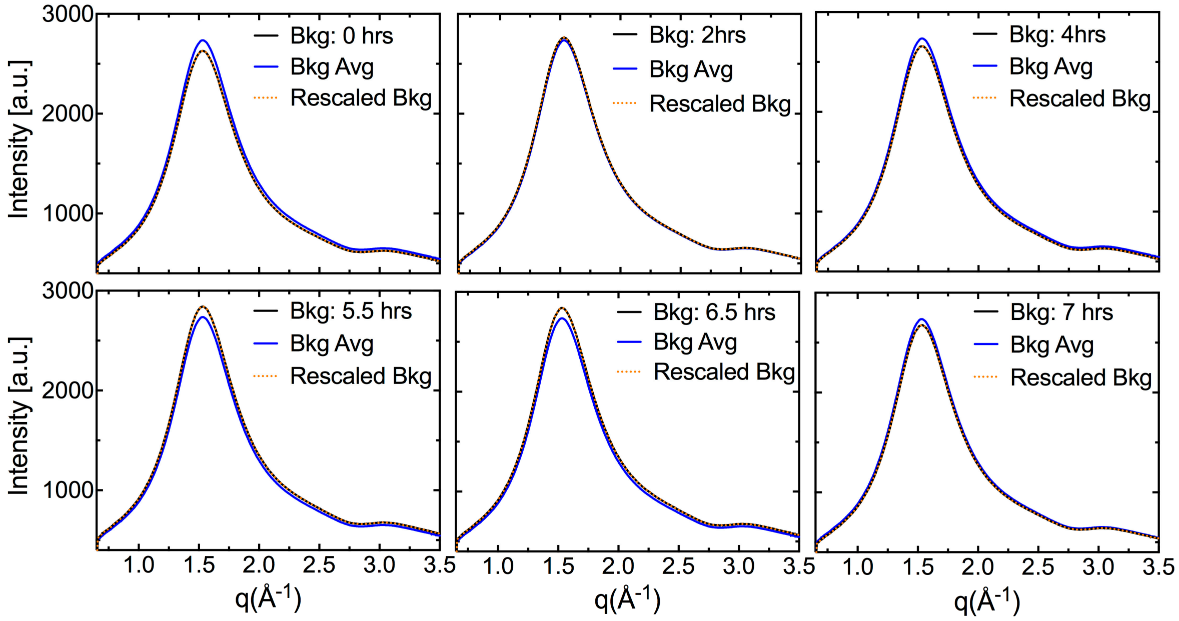

Figure S10: 1D WAXS profiles indicate how the average Pyrex background (blue) is rescaled (dashed golden line) to mimic each of the individual Pyrex frames.

Summary of *in situ* WAXS and SAXS data for T^e_{mold} = 80°C during the heating and annealing steps

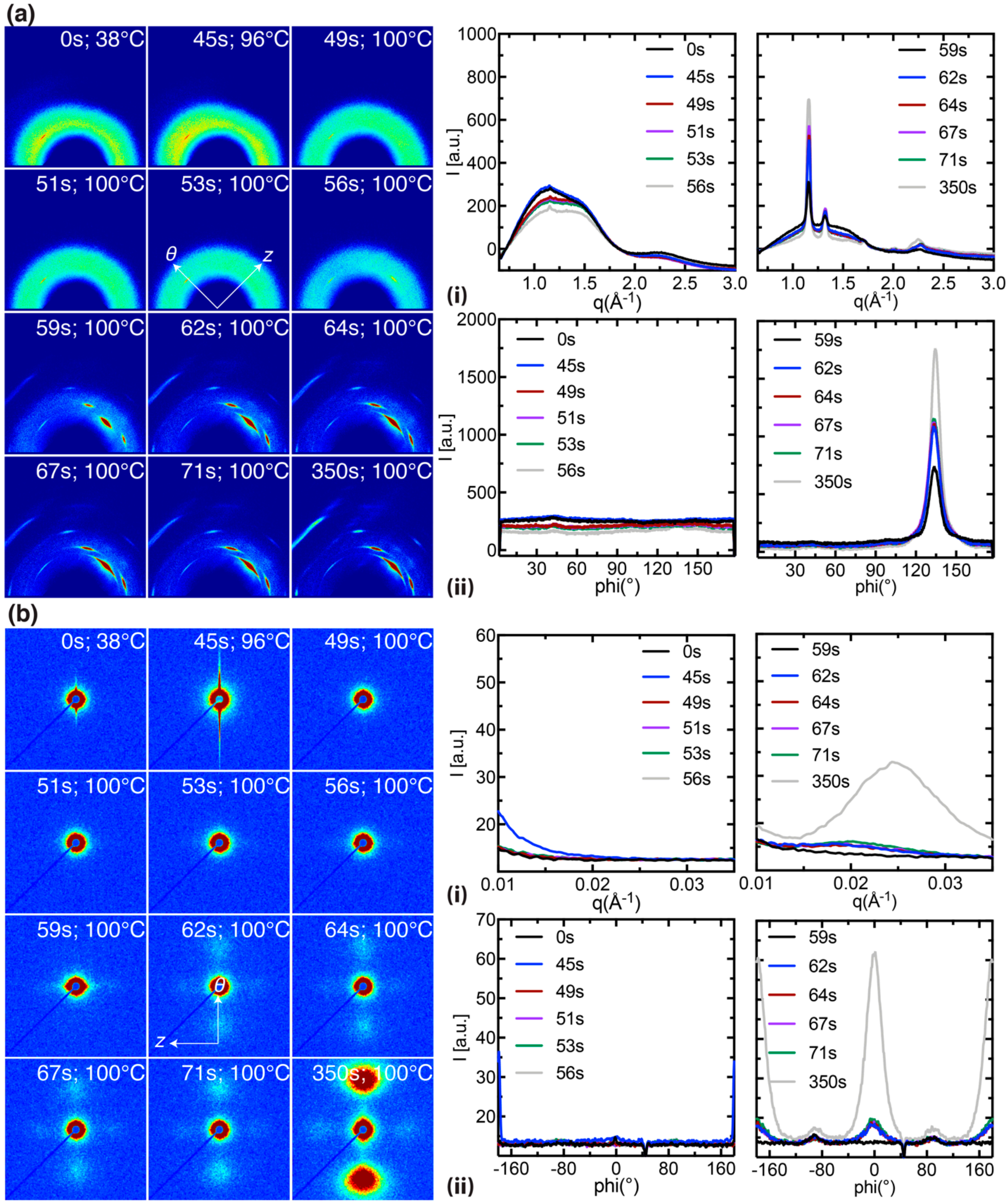

Figure S11: *In situ* **(a)** WAXS and **(b)** SAXS data acquired on a PLLA preform stretched at T^e_{mold} = 80°C. The corresponding temperature and strain profiles are presented in Figs. 3-4a. The WAXS and SAXS data are presented as **(a-b, left)** 2D patterns, **(a-b, i)** azimuthally averaged intensity, I(q), and **(a-b, ii)** radially averaged intensity, I(φ).

Summary of *in situ* WAXS and SAXS data for T^{e}_{mold} = 90°C during the heating and annealing steps

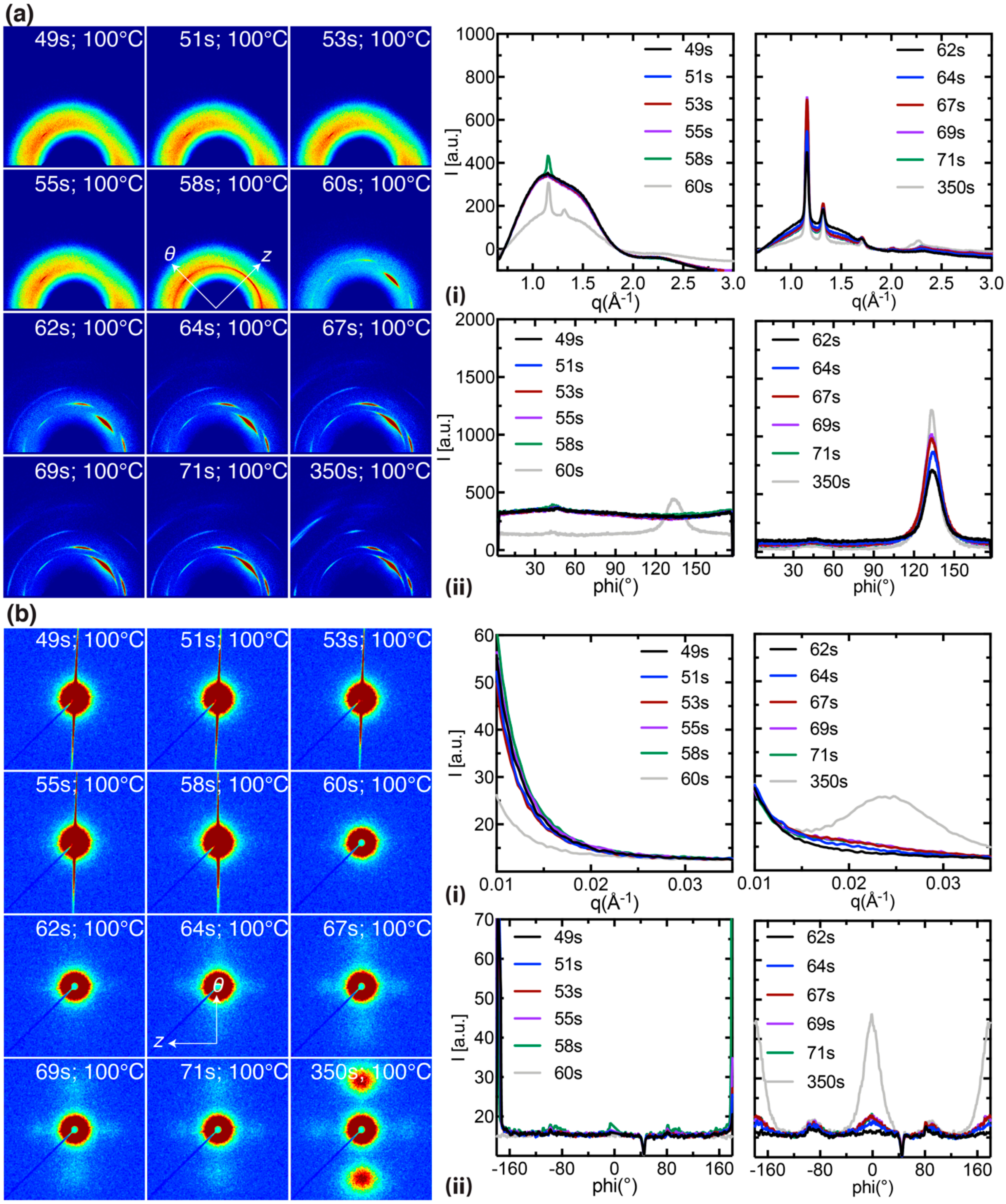

Figure S12: *In situ* **(a)** WAXS and **(b)** SAXS data acquired on a PLLA preform stretched at T^{e}_{mold} = 90°C. The corresponding temperature and strain profiles are presented in Figs. 3-4a. The WAXS and SAXS data are presented as **(a-b, left)** 2D patterns, **(a-b, i)** azimuthally averaged intensity, I(q), and **(a-b, ii)** radially averaged intensity, I(φ).

Summary of *in situ* WAXS and SAXS data for T^{x}_{mold} = 80°C during the heating and annealing steps

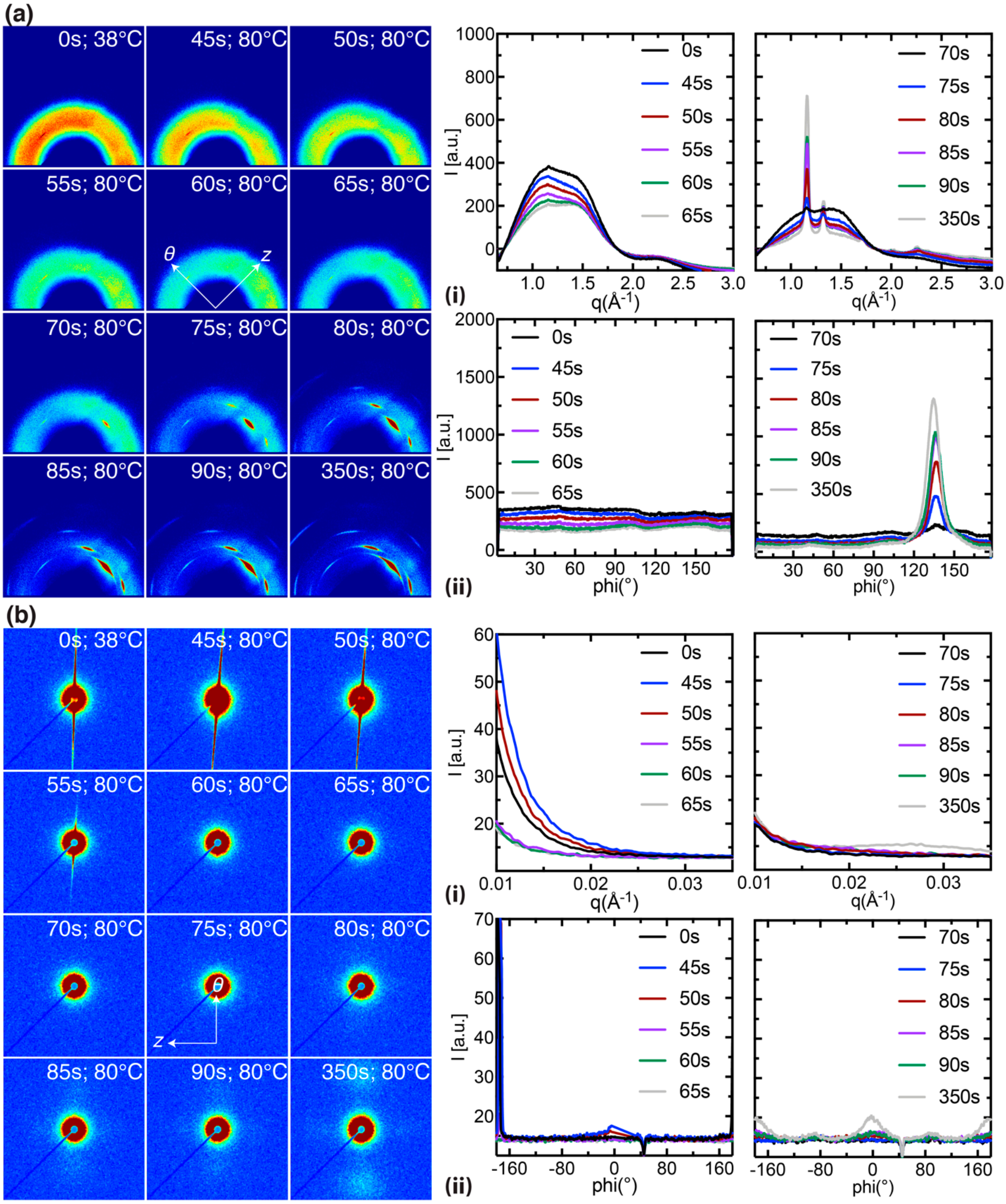

Figure S13: *In situ* **(a)** WAXS and **(b)** SAXS data acquired on a PLLA preform stretched at T^{x}_{mold} = 80°C. The corresponding temperature and strain profiles are presented in Figs. 3-4b. The WAXS and SAXS data are presented as **(a-b, left)** 2D patterns, **(a-b, i)** azimuthally averaged intensity, I(q), and **(a-b, ii)** radially averaged intensity, I(φ).

Summary of *in situ* WAXS and SAXS data for T^x_{mold} = 90°C during the heating and annealing steps

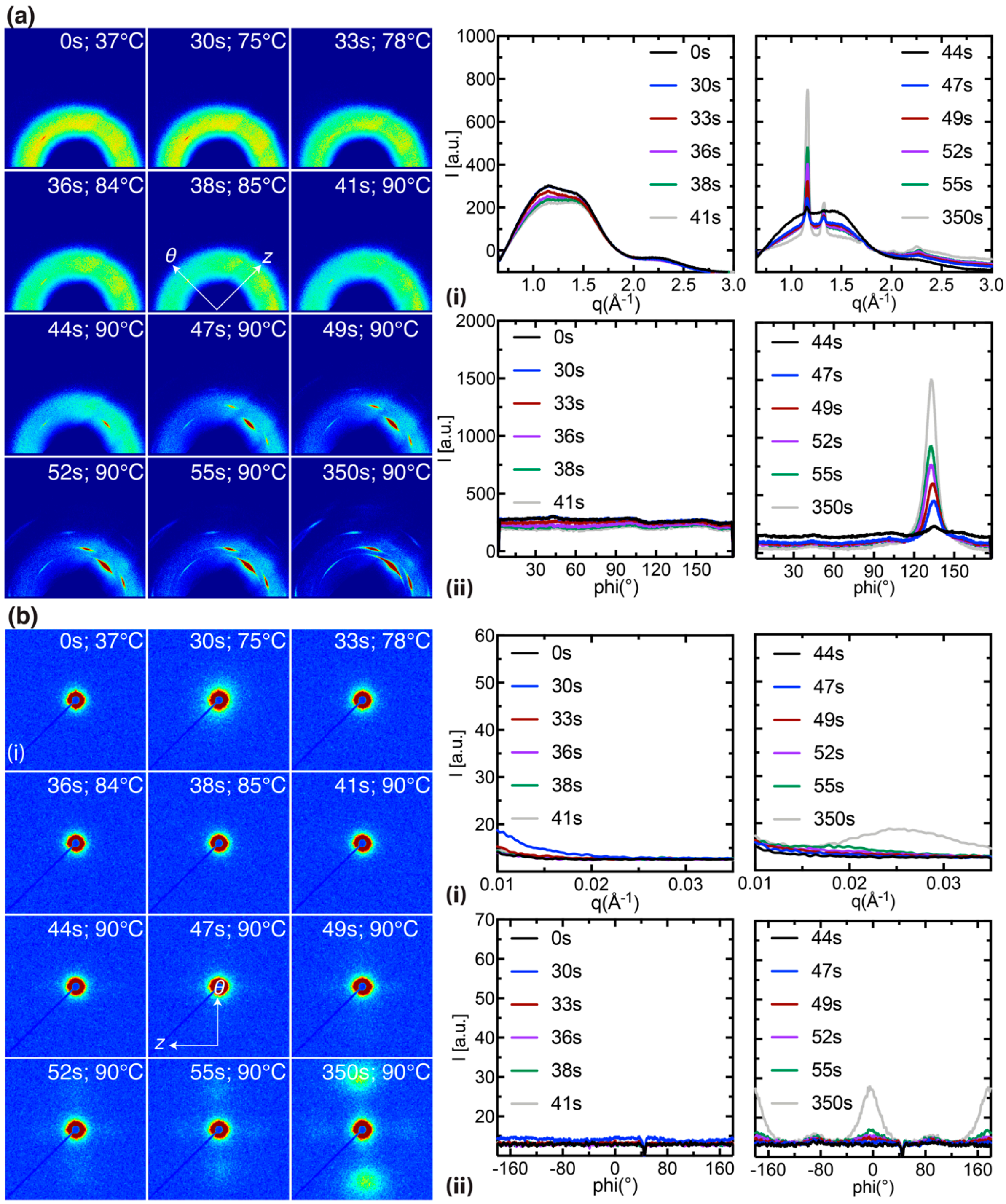

Figure S14: *In situ* **(a)** WAXS and **(b)** SAXS data acquired on a PLLA preform stretched at T^x_{mold} = 90°C. The corresponding temperature and strain profiles are presented in Figs. 3-4b. The WAXS and SAXS data are presented as **(a-b, left)** 2D patterns, **(a-b, i)** azimuthally averaged intensity, I(q), and **(a-b, ii)** radially averaged intensity, I(φ).

X-ray scattering data during the cooling phase

The intensity of the SAXS peaks increases during the first half of annealing (<200s Figs S15-20b) but decreases rapidly during cooling (>350s, Figs. S15-20b). On the other hand, the interlamellar spacing monotonically decreases with time post expansion during both the annealing and the cooling steps (Fig. S22a-b, right). We hypothesize that a combination of oriented crystallization and changes in density driven by temperature explain the observed trend in the SAXS data. The inflation of the tube imposes strains in excess of 400% at the inner diameter, which is likely to induce "shish-kebabs" along the θ-direction of the tube (meridional peaks in the SAXS patterns, Figs. S15-20b). This morphology is characterized by regularly spaced lamellar stacks called "kebabs" that decorate a central stem of oriented polymer chains called "shish". The SAXS intensity increases during the first 200s due to a decrease in the density of the interlamellar space and to the growth of kebabs; the gradual thickening of kebabs during annealing may explain the steady decrease in the long period. During the cooling step, the SAXS intensity decreases due to the densification of amorphous interlamellar space (see Fig. S15-20b); the possible growth of fringed micelles in the interlamellar space during cooling may also contribute towards a decrease in SAXS intensity. In the final SAXS frame, there is little to evidence of regularly alternating lamellar superstructures in the expanded tube (700s, Figs. S15-20b). Therefore, the transient structure reveals aspects of the PLLA semicrystalline morphology that are concealed in the final state of the expanded tube. The instrument also helps to identify expansion conditions that may favor the growth of shish-kebabs (e.g. the transient SAXS intensity is much stronger in T^{x}_{mold} = 100°C compared to T^{x}_{mold} = 80°C, compare Fig. S18 with Fig. S20), which may be advantageous for the BVS as they are known to boost strength in polymers by an order of magnitude.

Summary of *in situ* WAXS and SAXS data for T^{e}_{mold} = 80°C including the cooling phase

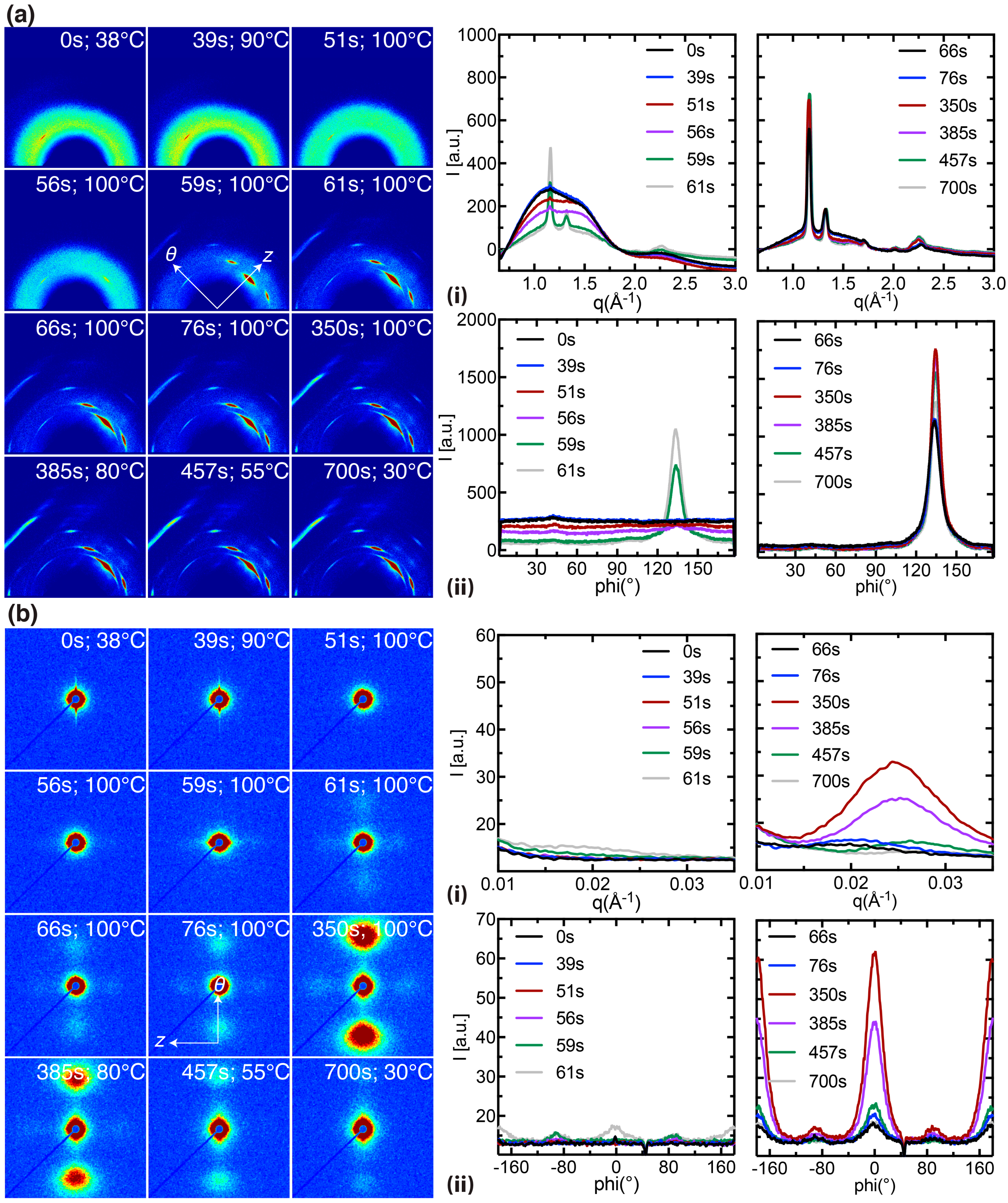

Figure S15: *In situ* **(a)** WAXS and **(b)** SAXS data acquired on a PLLA preform stretched at T^{e}_{mold} = 80°C; the first 9 patterns capture the transient expansion and annealing steps (t <350s, T^{a}_{mold} = 100°C) while the last 3 patterns capture the structure of the expanded tube during cooling (t > 350s). The WAXS and SAXS data are presented as 2D diffraction patterns, **(a-b, i)** azimuthally averaged intensity, I(*q*), and **(a-b, ii)** radially averaged intensity, I(φ).

Summary of *in situ* WAXS and SAXS data for T^{e}_{mold} = 90°C including the cooling phase

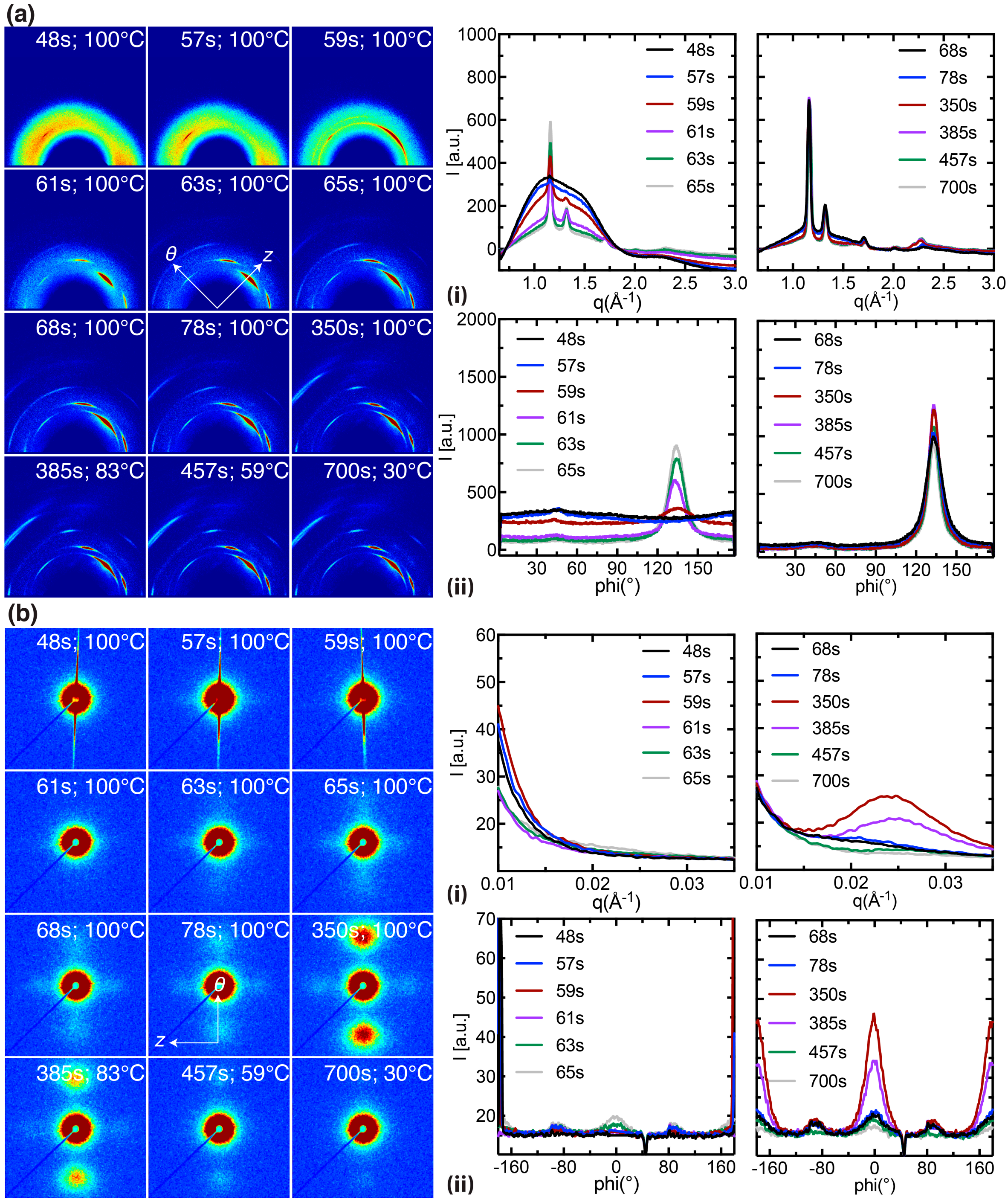

Figure S16: *In situ* **(a)** WAXS and **(b)** SAXS data acquired on a PLLA preform stretched at T^{e}_{mold} = 90°C; the first 9 patterns capture the transient expansion and annealing steps (t <350s, T^{a}_{mold} = 100°C) while the last 3 patterns capture the structure of the expanded tube during cooling (t > 350s). The WAXS and SAXS data are presented as 2D diffraction patterns, **(a-b, i)** azimuthally averaged intensity, I(q), and **(a-b, ii)** radially averaged intensity, I(φ).

Summary of *in situ* WAXS and SAXS data for T^e_{mold} = 100°C including the cooling phase

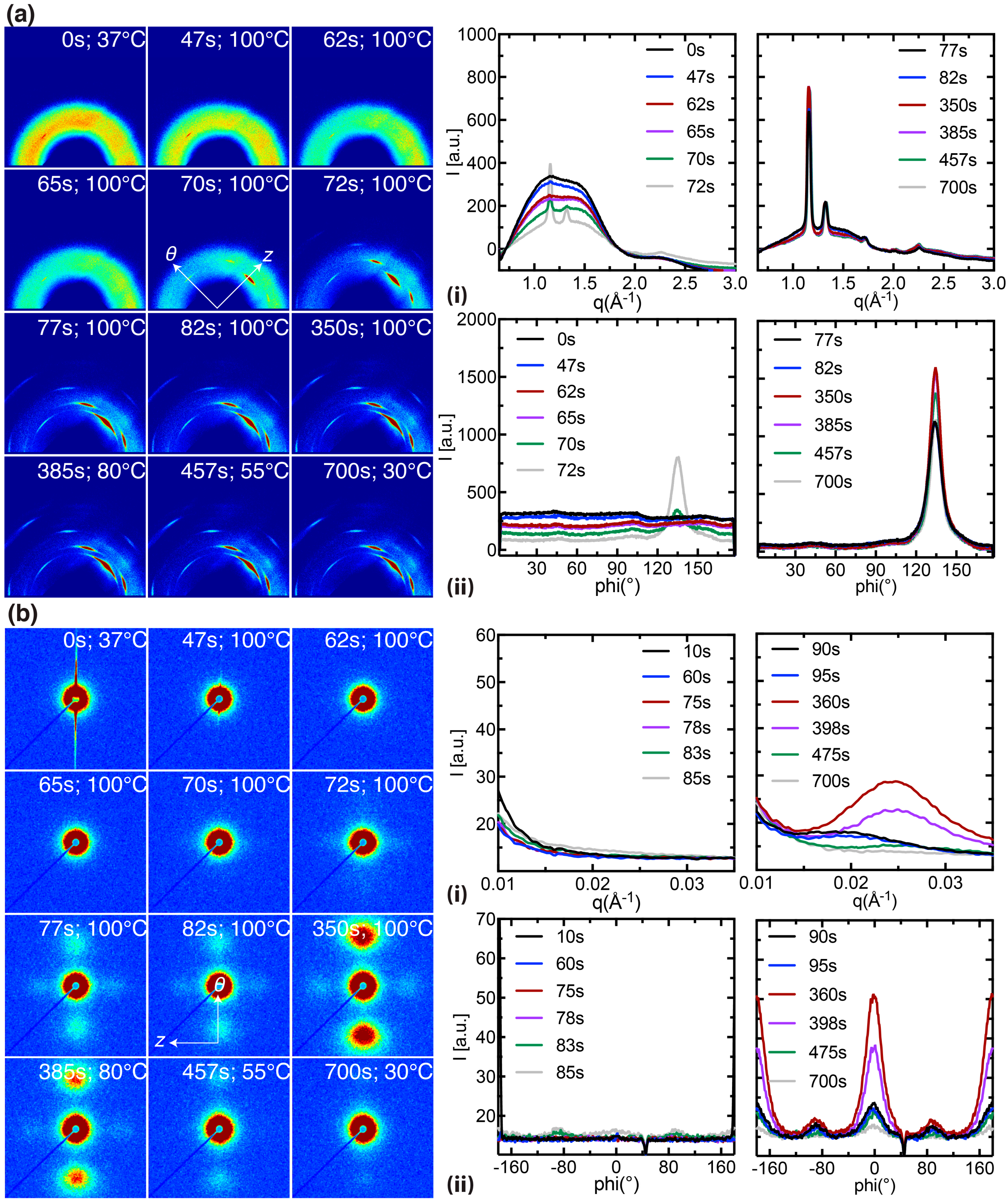

Figure S17: *In situ* **(a)** WAXS and **(b)** SAXS data acquired on a PLLA preform stretched at T^e_{mold} = 100°C; the first 9 patterns capture the transient expansion and annealing steps (t <350s, T^a_{mold} = 100°C) while the last 3 patterns capture the structure of the expanded tube during cooling (t > 350s). The WAXS and SAXS data are presented as 2D diffraction patterns, **(a-b, i)** azimuthally averaged intensity, I(*q*), and **(a-b, ii)** radially averaged intensity, I(φ).

Summary of *in situ* WAXS and SAXS data for T^{x}_{mold} = 80°C including the cooling phase

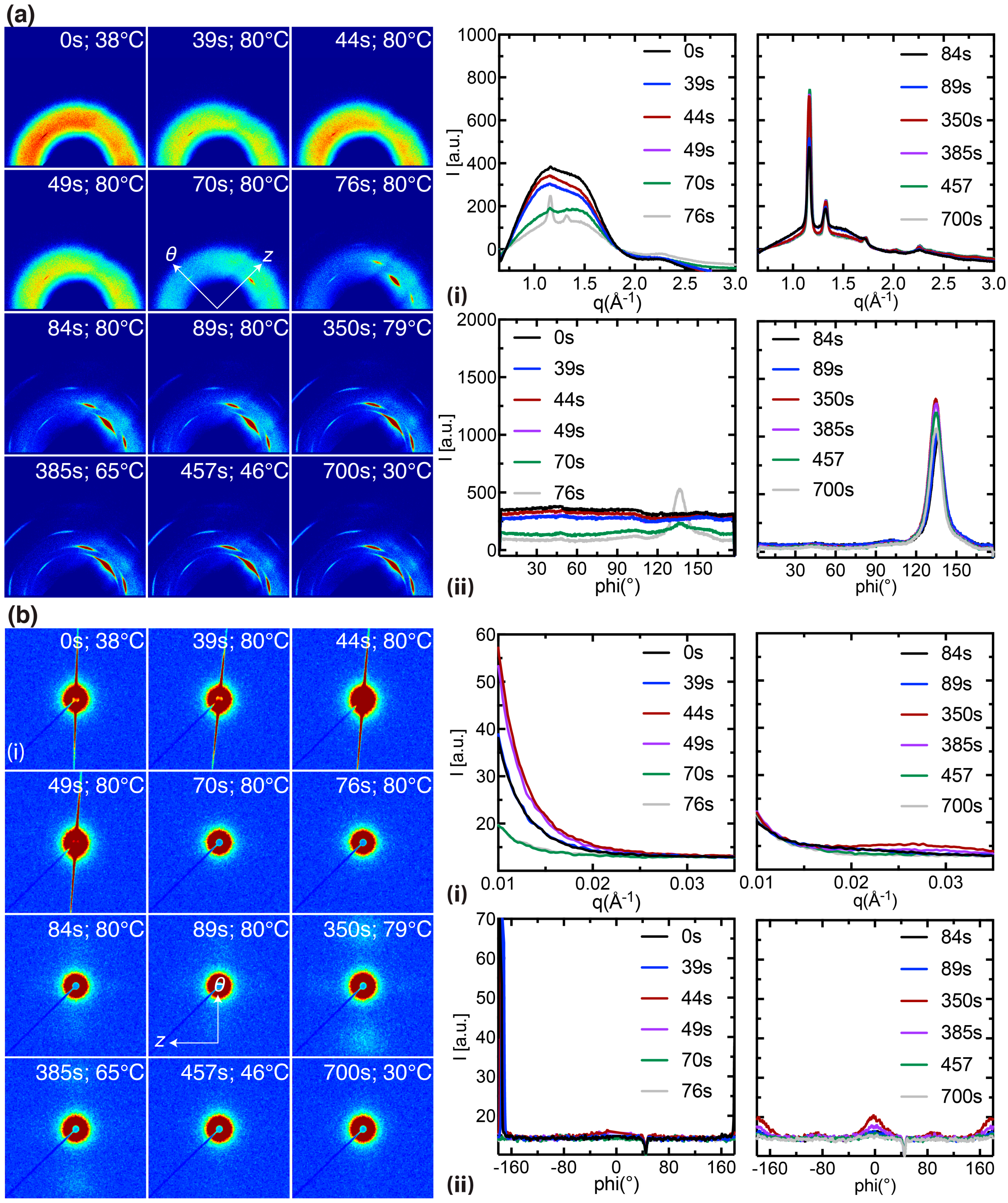

Figure S18: *In situ* **(a)** WAXS and **(b)** SAXS data acquired on a PLLA preform stretched at T^{x}_{mold} = 80°C; the first 9 patterns capture the transient expansion and annealing steps (t <350s, T^{a}_{mold} = 80°C) while the last 3 patterns capture the structure of the expanded tube during cooling (t > 350s). The WAXS and SAXS data are presented as 2D diffraction patterns, **(a-b, i)** azimuthally averaged intensity, I(*q*), and **(a-b, ii)** radially averaged intensity, I(φ).

Summary of *in situ* WAXS and SAXS data for T^{x}_{mold} = 90°C including the cooling phase

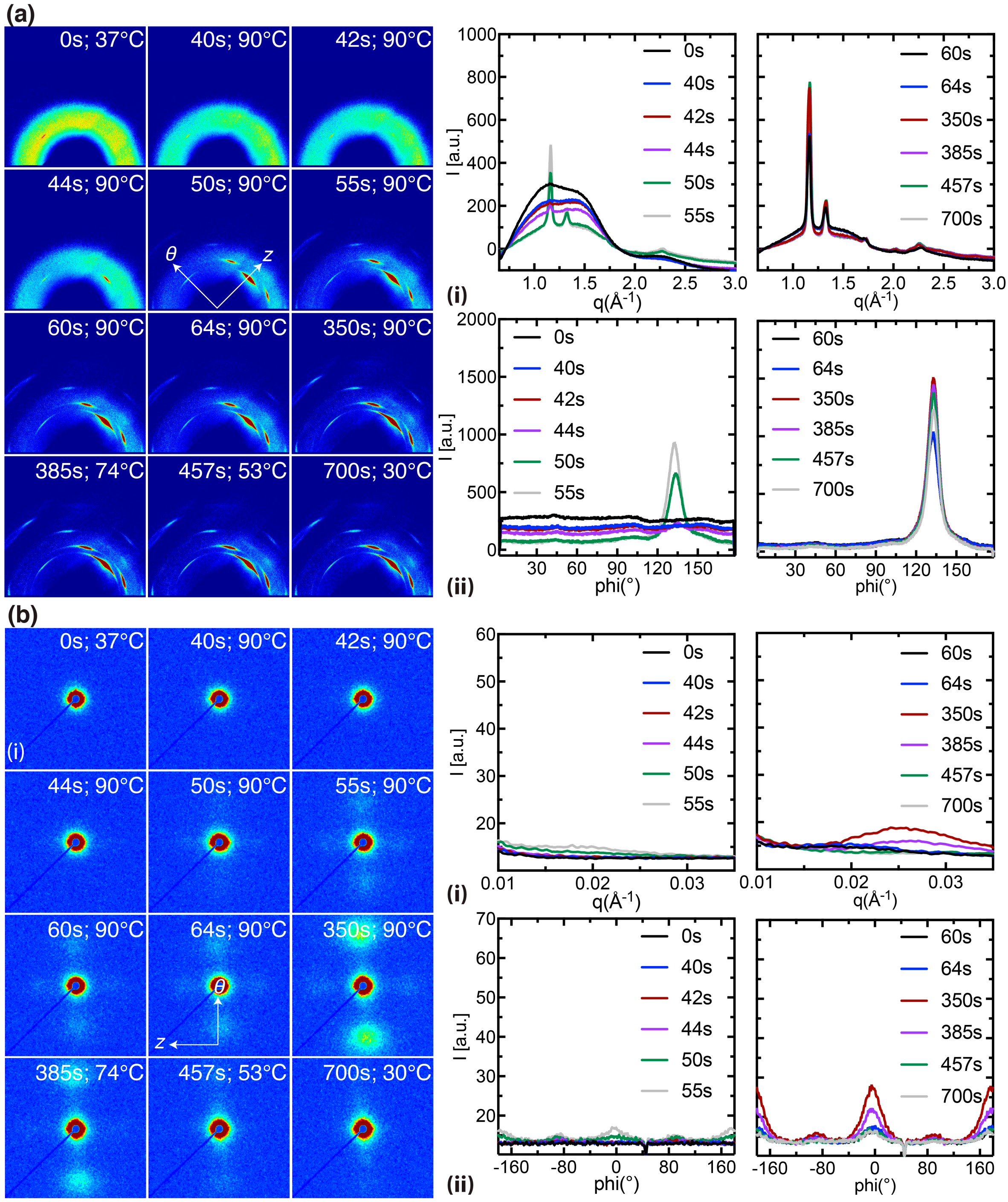

Figure S19: *In situ* **(a)** WAXS and **(b)** SAXS data acquired on a PLLA preform stretched at T^{x}_{mold} = 90°C; the first 9 patterns capture the transient expansion and annealing steps (t <350s, T^{a}_{mold} = 90°C) while the last 3 patterns capture the structure of the expanded tube during cooling (t > 350s) The WAXS and SAXS data are presented as 2D diffraction patterns, **(a-b, i)** azimuthally averaged intensity, I(q), and **(a-b, ii)** radially averaged intensity, I(φ).

Summary of *in situ* WAXS and SAXS data for T^{x}_{mold} = 100°C including the cooling phase

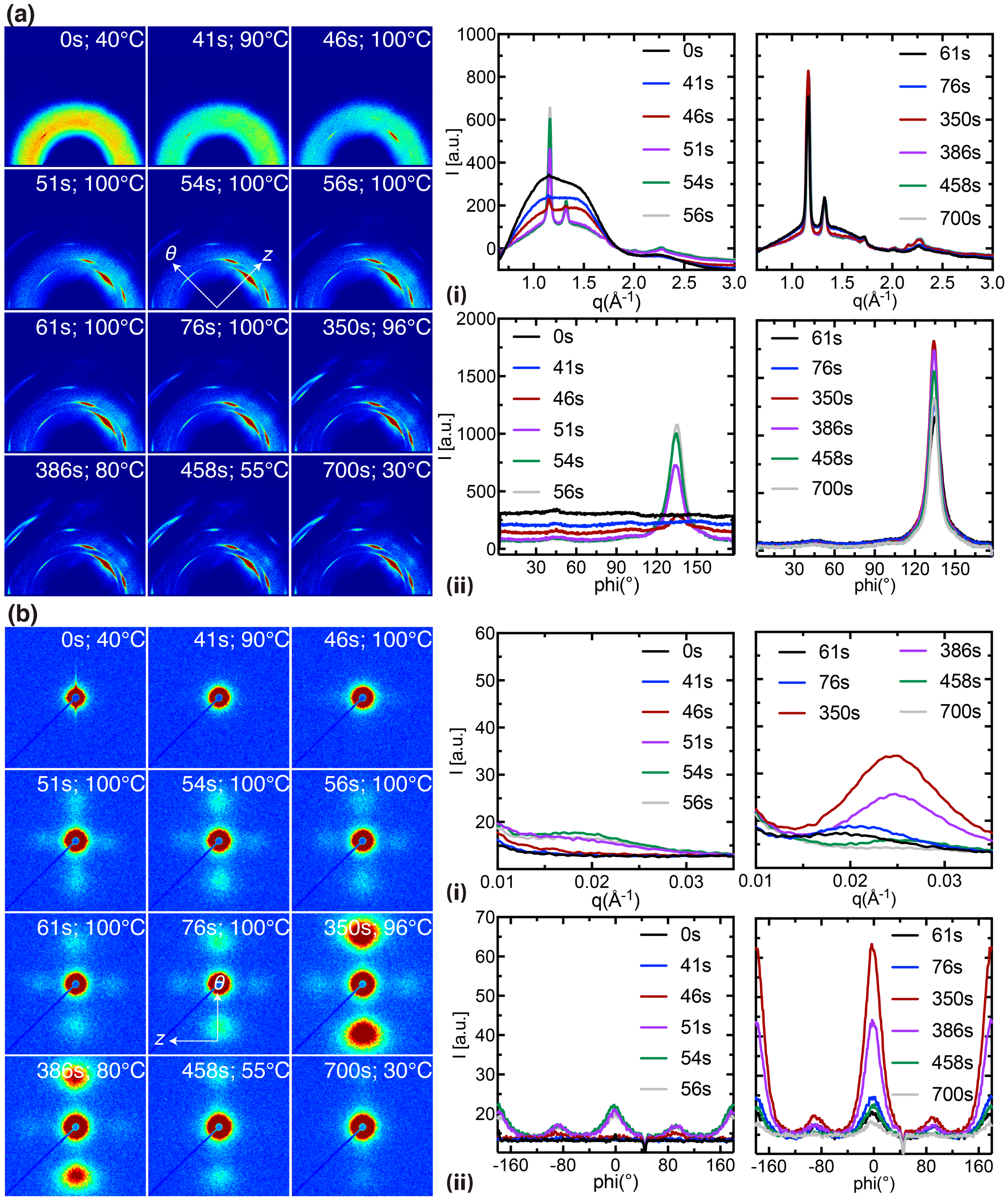

Figure S20: *In situ* **(a)** WAXS and **(b)** SAXS data acquired on a PLLA preform stretched at T^{x}_{mold} = 100°C; the first 9 patterns capture the transient expansion and annealing steps (t <350s, T^{a}_{mold} = 100°C) while the last 3 patterns capture the structure of the expanded tube during cooling (t > 350s) The WAXS and SAXS data are presented as 2D diffraction patterns, **(a-b, i)** azimuthally averaged intensity, I(q), and **(a-b, ii)** radially averaged intensity, I(φ).

Quantitative WAXS analysis for patterns acquired during the heating, annealing and cooling steps

The *q*-position of the (110)/(200) peaks is shifted towards lower values during annealing in accord with thermal expansion of the crystal lattice (t < 350s, Fig. S21c); at the end of the cooling step, it appears that the crystallites predominantly belong to the rigid and ordered α morph (q ~ 1.17 Å^{-1}, Fig. S21) [5–7].

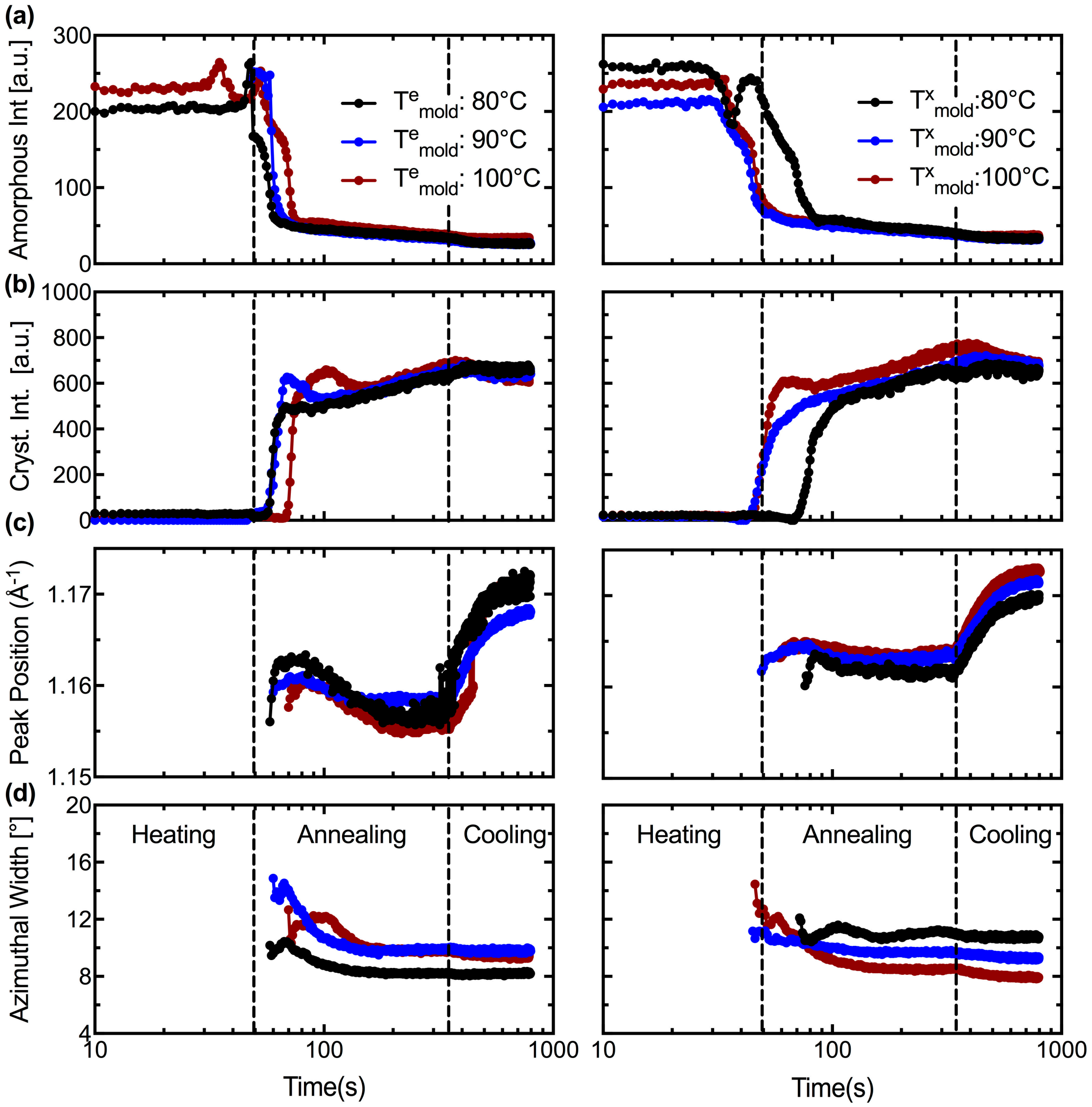

Figure S21: Quantitative characteristics of 1D WAXS profiles for expansion performed at **(left)** T^{e}_{mold} = 80, 90 and 100°C and **(right)** T^{x}_{mold} = 80, 90 and 100°C. The variation in (**a**) amorphous content, (**b**) crystallinity, (**c**) peak position of the (110)/(200) peaks and (**d**) full width at half maximum of the (110)/(200) peaks is presented. The vertical dashed lines indicate the onset of the annealing and cooling steps. The corresponding temperature profiles are presented in Figure 3 of the main text.

Quantitative SAXS analysis for data acquired during the heating, annealing and cooling steps

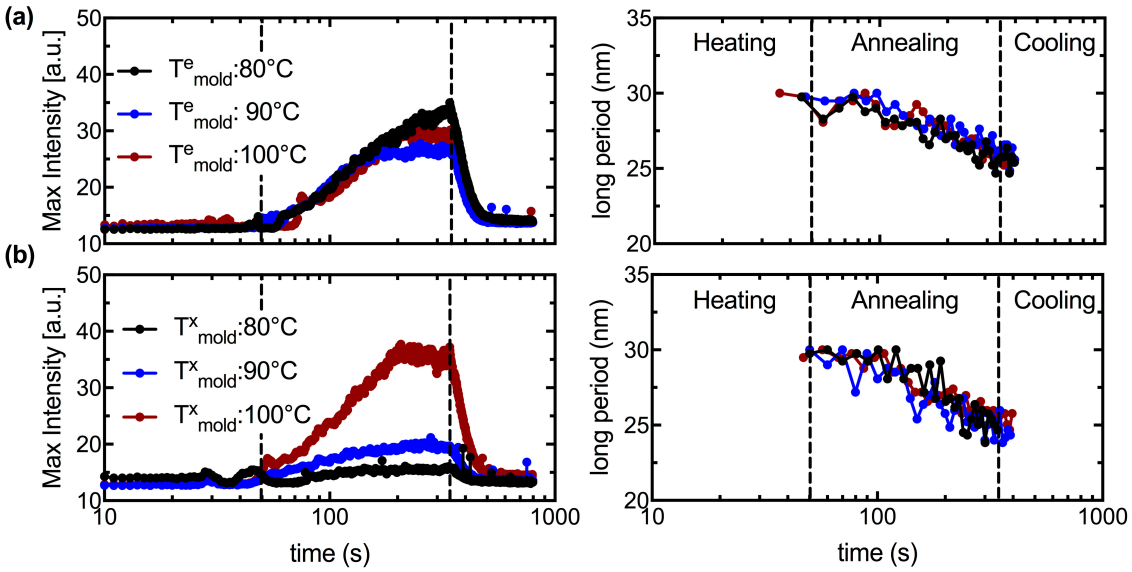

Figure S22: Quantitative characteristics of 1D SAXS profiles for expansion performed at **(a)** T^{e}_{mold} = 80, 90 and 100°C and **(b)** T^{x}_{mold} = 80, 90 and 100°C. The variation in **(a-b, left)** maximum intensity of the meridional SAXS peaks and **(a-b, right)** the interlamellar spacing is presented. The vertical dashed lines indicate the onset of the annealing and cooling steps. The corresponding temperature profiles are presented in Figure 3 of the main text.

References:

1. Mulbry, W.; Reeves, J. B.; Millner, P. Use of mid- and near-infrared spectroscopy to track degradation of bio-based eating utensils during composting. *Bioresour. Technol.* **2012**, *109*, 93–97, doi:10.1016/j.biortech.2012.01.029.
2. Schnelle, W.; Engelhardt, J.; Gmelin, E. Specific heat capacity of Apiezon N high vacuum grease and of Duran borosilicate glass. *Cryogenics* **1999**, *39*, 271–275, doi:10.1016/S0011-2275(99)00035-1.
3. Aou, K.; Kang, S.; Hsu, S. L. Morphological Study on Thermal Shrinkage and Dimensional Stability Associated with Oriented Poly (lactic acid). *Macromolecules* **2005**, *38*, 7730–7735.
4. Kim, H. S.; Chae, Y. S.; Park, B. H.; Yoon, J. S.; Kang, M.; Jin, H. J. Thermal and electrical conductivity of poly(l-lactide)/multiwalled carbon nanotube nanocomposites. *Curr. Appl. Phys.* **2008**, *8*, 803–806, doi:10.1016/j.cap.2007.04.032.
5. Di Lorenzo, M. L.; Cocca, M.; Malinconico, M. Crystal polymorphism of poly(l-lactic acid) and its influence on thermal properties. *Thermochim. Acta* **2011**, *522*, 110–117, doi:10.1016/j.tca.2010.12.027.
6. Wasanasuk, K.; Tashiro, K.; Hanesaka, M.; Ohhara, T.; Kurihara, K.; Kuroki, R.; Tamada, T.; Ozeki, T.; Kanamoto, T. Crystal structure analysis of poly(l-lactic acid) α form on the basis of the 2-dimensional wide-angle synchrotron X-ray and neutron diffraction measurements. *Macromolecules* **2011**, *44*, 6441–6452, doi:10.1021/ma2006624.
7. Billimoria, K.; Heeley, E. L.; Parsons, N.; Figiel, Ł. An investigation into the crystalline morphology transitions in poly-L- lactic acid (PLLA) under uniaxial deformation in the quasi-solid-state regime. *Eur. Polym. J.* **2018**, *101*, 127–139, doi:S0014305717322292.
